# Building dynamical models of multi-step state transitions from single cell gene expression trajectories

**DOI:** 10.64898/2025.12.08.693064

**Published:** 2025-12-11

**Authors:** Yukai You, Cristian Caranica, Mingyang Lu

**Affiliations:** 1 Center for Theoretical Biological Physics, Northeastern University, Boston, MA 02115, USA; 2 Department of Bioengineering, Northeastern University, Boston, MA 02115, USA

**Keywords:** GRN inference, dynamic models, cell state transition, gene regulatory circuits, iPSC differentiation

## Abstract

Multi-step cell state transitions often occur in biological processes, such as cell differentiation and disease progression, yet the regulatory mechanisms governing these transitions remain unclear. Here, we introduce NetDes, a computational method that integrates top-down and bottom-up systems biology to infer core transcription factor regulatory networks and build ODE-based dynamical models from single-cell gene expression trajectories. We demonstrate that NetDes predicts regulatory interactions and reproduces gene expression dynamics through benchmarking using in-silico time trajectories with decoys, tests on gene circuit simulations of embryonic phenotypic switching, and application to time-series scRNA-seq data from human iPSC differentiation. Compared to existing approaches, NetDes has the advantage of capturing sequential state transitions within a single dynamical model. Network simulations and coarse-graining further elucidate the regulatory roles of genes and their combinations in driving these transitions. Our approach provides a generalizable framework for mechanistic modeling of gene regulation in complex cell state transitions.

## Introduction

Cell state transitions play a crucial role in many biological processes, such as embryonic development^1–3^, tissue regeneration^4,5^, and disease progression^6,7^. Technological advances in genomics^8–10^ and live-cell imaging^11^ have revealed that cell state transitions often proceed gradually through multiple steps of intermediate states, in contrast to binary state switches. For example, cancer cells can undergo a complete Epithelial-Mesenchymal transition (EMT) by transiting through hybrid states^12^. During the development of hematopoietic stem cells (HSCs), progenitor cells pass through pre-HSC stages before becoming fully functional^13^. In mouse embryonic stem cell (mESC) differentiation, the formative state has been identified as an intermediate state between the naive and primed states^3^. Elucidating the regulatory principles underlying multi-step state transitions remains a major question in systems biology.

Single-cell RNA sequencing (scRNA-seq) has become a widely used technology for studying cell state transitions, as it enables genome-wide gene expression profiling of individual cells undergoing these transitions. With advances in computational methods, researchers can now map dynamical changes in cell states from scRNA-seq data. For example, pseudotime inference^14,15^ and lineage tracing^9^ approaches allow cells to be ordered along continuous developmental trajectories, while methods related to RNA velocity^16,17^ enable the prediction of single-cell vector fields to represent gene expression dynamics. However, while these approaches help to describe state transitions, it is still challenging to elucidate the gene regulation of these transitions and accurately predict key regulators capable of driving specific transitions.

Many computational methods have been developed to infer gene regulatory networks (GRNs) from scRNA-seq data, using statistical inference^18–21^, machine learning^22–24^, or dynamical systems approach^16,18,25–27^. Despite these advances, most existing methods focused on reconstruction of large networks for individual cell types or discrete cell states, rather than modeling regulatory systems that drives continuous multi-step transitions. For example, scMTNI employs a multi-task learning framework to infer GRNs along a defined lineage, where discrete GRNs are built for individual cell states^22^. LINGER uses a neural-network-based method to reconstructs either a cell population GRN, cell-type-specific GRNs or cell-level GRNs^24^; however, the method was not designed to construct a GRN capturing state transition dynamics. Furthermore, inferring causal regulatory interactions directly from statistical associations proves to be difficult^28,29^, especially when analyzing noisy single-cell datasets that include technical noise such as dropout effects^30^ and biological noise derived from stochastic gene expressions^31^.

In our view, these limitations could be alleviated by the following strategy that integrates top-down and bottom-up systems biology^32^. First, a relatively small set of genes, known as transcription factors (TFs), largely regulate the transcription of the other genes, including other TFs, although exceptions do exist. The regulatory interactions among a set of core TFs may form functional gene circuits that perform specific cellular functions, such as gene expression oscillators in the cell cycle^33^ and multistable switches governing cell fate determination^34^. Thus, we aim to model a core set of TFs and the regulatory interactions among them and evaluate how effectively such core TF networks can capture the observed gene expression dynamics during cell state transitions. Second, to improve the mechanistic inference from noisy single cell data, we incorporate literature-bwased candidate interactions and denoise the data by smoothing gene expression trajectories along pseudotime, which generates much cleaner inputs for constructing minimal ordinary differential equation (ODE)-based models of a GRN. Finally, based on the optimized mathematical models, subsequent systems biology analyses, such as network simulations of extrinsic signaling and network coarse-graining^35,36^, could uncover the dynamical properties of the GRNs. Such insights into network dynamics are often difficult to obtain using conventional network inference methods alone. In the following, we will present the computational method and demonstrate its utility through benchmarking on both synthetic datasets and experimental scRNA-seq data.

## Results

### Overview of NetDes

**Fig.1.**
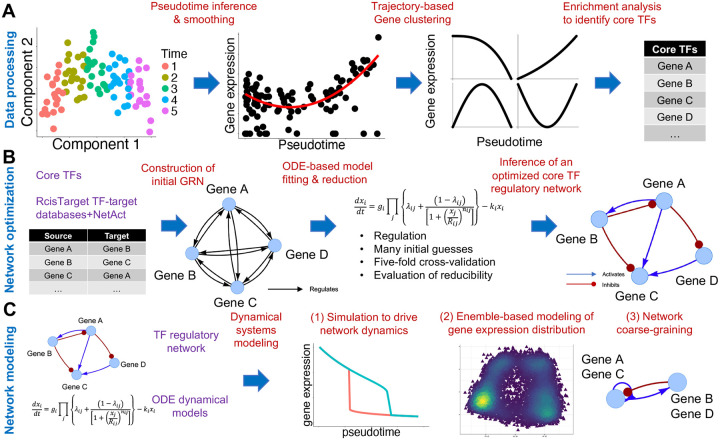
Overview of NetDes for inferring dynamical models of GRN using scRNA-seq data. **(A)** Data processing. From time-series scRNA-seq data, we first infer pseudotime with a higher time resolution. We then apply PseudotimeDE to fit a smooth time trajectory for each gene. Genes are then clustered according to the shapes of their time trajectories. From the gene clusters, we identify core transcription factors (TFs) whose target genes are enriched in the time trajectory clusters. **(B)** Network optimization. An initial gene regulatory network (GRN) is built by connecting the inferred core TFs with regulatory interactions from literature-based TF-target databases. From the initial GRN, we optimize a minimal GRN and the associated nonlinear ODE-based dynamical model to match gene expression time trajectories. **(C)** Network modeling. With the established GRN model, we can perform (1) simulations to drive cell state transitions with extrinsic signals to network genes; (2) ensemble-based modeling to explore robust network states; (3) network coarse-graining to infer core gene circuits.

We introduce NetDes, a computational method for constructing core TF regulatory networks that drive multi-step cell state transitions and building dynamical models using gene expression time trajectories. The workflow of NetDes is outlined in Fig.1. First, from time-series scRNA-seq data (the leftmost panel in Fig.1A), NetDes infers pseudotime with a higher time resolution. Second, for each gene, gene expression time trajectory is fitted and smoothed along inferred pseudotime (2^nd^ panel from the left in Fig.1A). This step is achieved by applying PseudotimeDE^37^, which models each cell’s gene expression as sampled from a negative binomial distribution, where the mean expression along pseudotime is fitted by a spline curve. Third, these genes are clustered according to the shapes of the gene expression time trajectories (3^rd^ panel from the left in Fig.1A). Fourth, each context-specific TF is identified whose target genes are enriched in a trajectory cluster (the rightmost panel in Fig.1A). This enrichment analysis is performed by assuming that genes with similar time trajectories are likely regulated by the same TF. Fifth, NetDes constructs an initial GRN of the enriched TFs by connecting the genes with regulatory links according to the TF-target databases in TRRUST^38^ and the TF binding motifs identified using Rcistarget^39^ (1^st^ and 2^nd^ panels in Fig.1B). At this stage, the activating/inhibiting nature of regulatory interactions are yet to be determined. Sixth, nonlinear ODEs are fitted against gene expression time trajectories, allowing to trim unnecessary interactions, as well as constructing an ODE-based dynamical model of the optimized GRN (3^rd^ and 4^th^ panels in Fig.1B). The established dynamical model also specifies whether a regulatory link is excitatory or inhibitory. Finally, using the established dynamic models, we can perform dynamical systems modeling, including gene expression dynamics simulations for the GRN driven by extrinsic signaling, ensemble-based modeling of gene expression distribution from the reconstructed GRN, and network coarse-graining (Fig.1C). The step-by-step procedure for NetDes is detailed in the Methods section.

A major component of NetDes is a robust algorithm for fitting and optimizing a GRN and the associated nonlinear ODE model (the sixth step mentioned above). For every network gene, the kinetic parameters for the gene’s ODE are fitted according to the regulators’ and target’s smoothed gene expression trajectories by a four-stage model optimization (see Methods and Fig.S1 for details). To prevent the nonlinear fitting from being trapped in local minima, NetDes performs model fitting over multiple rounds to sample different combinations of regulators’ activating and inhibiting types. To ensure robust model fitting, NetDes incorporates regularization terms, five-fold cross-validation (CV), and a model reduction scheme. Overall, these strategies ensure a reliable dynamical model generation of minimal GRNs capturing multi-step state transitions (see Fig.S2 for the workflow of the model optimization).

### Benchmarking with in-silico gene expression trajectories

**Fig.2.**
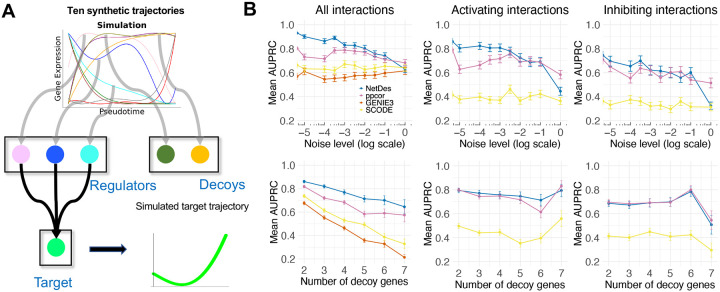
Benchmarking network inference using synthetic trajectories with decoys. **(A)** Illustration of the process used to generate the synthetic dataset for benchmarking. First, different gene expression time trajectories are generated for ten genes. Second, for each test case, a subset of these genes is randomly selected as regulators. Their gene expression trajectories are then used to simulate the trajectory of a target gene according to an ODE model with randomly selected kinetic parameters. Third, additional genes from the remaining set are chosen as decoys. Finally, in each test, the time trajectories of true regulators, target gene, and decoys are provided as input to network inference methods. The goal is to evaluate whether a network inference method can effectively distinguish true regulators from decoys. **(B)** Benchmarking results for the synthetic dataset. Top panels show the AUPRC of different network inference methods applied to simulated gene expression data under different noise levels. Bottom panels show the mean AUPRC of various methods when applied to cases with different numbers of decoy genes. The leftmost, middle and rightmost columns show the benchmark results for all interactions (i.e., both activating and inhibitory), activating interactions, and inhibitory interactions, respectively.

To evaluate the capacity of NetDes in identifying correct regulators of a target gene, we performed a comprehensive benchmarking on *in-silico* dataset against several popular network inference methods, *i.e*., PPCOR^20^, GENIE3^23^ and SCODE^26^ (Fig.2A). In this benchmark test, we randomly generated ten different time trajectories from polynomials (Eq. S1) to represent regulators’ expression dynamics (Fig.S3). For each case, a subset of randomly selected regulators (serving as ground truth) was assigned to a target gene, and the target gene’s expression dynamics were simulated using Eq. 12 with a set of randomly sampled kinetic parameters. Each test provides as the input the trajectories of the regulators and target, alongside the trajectories of randomly selected non-regulators (serving as decoys). From a total of 81 tests, we evaluated how various network inference methods can correctly identify the true regulators of the target gene (see Methods for details). From the benchmarking, we found NetDes outperforms the other methods in identifying the ground truth regulators according to both AUPRC (leftmost points in the top left panel in Fig.2B) and AUROC (Fig.S4). NetDes’s performance was robust in the benchmark test when noises of different levels were added to the gene expression time trajectories (top left panels in Fig.2B and Fig.S4). When evaluating the outcomes of the inference for activators and inhibitors separately, we found all methods performed slightly worse for predicting inhibitors, whereas NetDes still performed better (middle and right panels in Fig.2B and Fig.S4). Moreover, the performances of various methods became worse when testing on cases with more decoy genes, as these tests are likely more challenging (bottom panels in Fig.2B and Fig.S4).

### Reconstruction of the gene circuit governing ICM-TE transition

After testing NetDes on in-silico gene expression trajectories, we next evaluated the performance of NetDes in reconstructing a small gene regulatory circuit. The system we investigated is a three-node circuit, consisting of Oct4, Cdx2 and Esrrb, that governs earlier embryonic development in mouse. Oct4 maintains the inner cell mass (ICM) identity, whereas Cdx2 drives extraembryonic trophectoderm (TE) commitment. Around the early blastocyst, ICM cells retain the plasticity to reversibly switch to the TE fate but can become committed to the TE lineage through the activation of the FGF/MAPK signaling pathway^40^.

Here, we utilized this biological circuit to generate simulated gene expression trajectories and evaluated how well NetDes can reconstruct the circuit and recover its cell state transition properties through a dynamical model. To establish the ground-truth gene circuit (top left panel in Fig.S5), we conducted a manual literature survey to identify relevant gene regulatory interactions as follows. It has been shown that Esrrb actives Oct4 transcription in embryonic stem cells^41^, and Cdx2 represses Esrrb transcription in trophoblast stem cells according to ChIP-seq evidences^42^. Oct4 and Cdx2 form a toggle switch through mutual inhibition^43^, while the incorporation of Esrrb adds a coherent feedforward loop. We also included FGF as an input signaling node that drives the circuit directly through Cdx2^40^.

To generate the simulated dataset for gene circuit reconstruction, we establish an ODE model which allows two stable steady states corresponding to the ICM and TE states, respectively. The detailed ODEs and model parameters are provided in Table.S1. We then simulated the expression dynamics of Oct4, Cdx2 and Esrrb by driving the circuit from the ICM state to the TE state through an increasing FGF signal (bottom left panel in Fig.S5). For network modeling tests, we applied NetDes twice on two initial networks: the first was a fully connected network, while the second lacked the interaction from Oct4 to Esrrb, making it closer to the ground truth. In both cases, the simulated gene expression trajectories of all genes were inputted to NetDes.

From circuit reconstruction, NetDes recovered the ground truth circuit when starting from the second initial GRN, whereas it inferred an additional activation from Oct4 to Esrrb when starting from the first initial GRN. When simulating the optimized GRN models, we found, in both cases, FGF successfully drove the system in both directions. However, only in the second case could the circuit dynamics be driven backward by directly activating Cdx2 (Fig.S6). These results demonstrate NetDes’s ability to optimize small gene circuits that drive cell state transitions, while its performance can depend on quality of the initial GRN.

### Modeling sequential cell state transitions in iPSC differentiation

So far, we have evaluated the performance of NetDes using synthetic benchmarking tests and a small biological gene circuit. Next, we will demonstrate its usage by applying NetDes, along with other existing methods, to experimental single-cell RNA-seq data. The goal of this application is to assess the ability of various network inference methods to accurately reconstruct GRNs, while ensuring the inferred networks can capture multi-step cell state transitions observed in the experimental scRNA-seq data.

**Fig.3.**
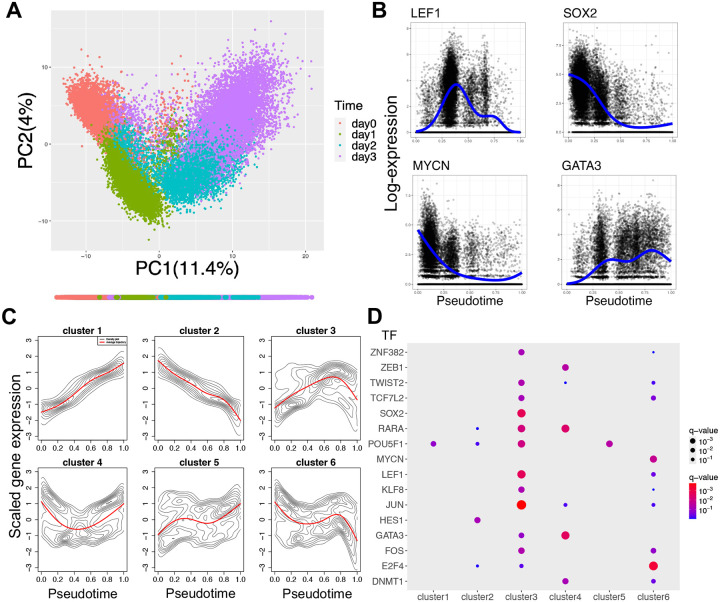
Gene expression trajectory inference using time series scRNA-seq data for iPSC to definitive endoderm differentiation. **(A)** Scatter plot showing the normalized expression levels of the top 500 highly variable genes (HVGs) projected onto the first two principal components (PCs). Different colors represent measured time points, as shown for each cell in the scatter plot, with the bottom color bar indicating pseudotime ordering inferred by Slingshot. **(B)** Examples of smoothed gene expression trajectories along the pseudotime (in blue dashed lines) for selected transcription factors (TFs), as inferred by PseudotimeDE using single cell expression levels (black dots). **(C)** Gene clustering on the top 5,000 HVGs based on the shape of their expression trajectories. Six distinct gene clusters were identified: two with monotonically increasing or decreasing trajectories, two with a single turning point (up-then-down in cluster 3; down-then-up in cluster 4), and two with two turning points. **(D)** Gene enrichment analysis using literature-based TF-target gene sets identifies significant TFs for each gene cluster (q-value < 0.1).

We analyzed a publicly available time-series scRNA-seq dataset of human induced pluripotent stem cell (iPSC) differentiation toward definitive endoderm (DE)^44^. In this experiment, iPSCs from 125 donors (Day 0) were treated with a cytokine-small-molecule cocktail to drive differentiation through mesendoderm (Day 1) to DE (Day3). Projection of the top 500 highly variable genes (HVGs) onto the first two principal components (PCs) reveals a clear path of continuous cell state transitions from iPSC to DE via mesendoderm (Fig.3A). This dataset is ideal for our testing because iPSC differentiation is well studied with extensive literature support, and it allows us to evaluate how well the inferred GRNs capture the multi-step state transitions.

Before NetDes is applied to build and optimize GRN models, initial data processing and statistical analysis are required to generate an initial GRN. We first inferred pseudotime using slingshot^45^ based on the top 500 HVGs and first five PCs. The inferred pseudotime aligns well with the observed cell state transitions (Fig. 3A). To mitigate inherently stochastic nature of gene expression in individual cells and technical noise, such as the dropout effects, (Fig. 3B) we applied PseuodtimeDE^37^ to generate smoothed gene expression time trajectories along the inferred pseudotime (details in the Method section, examples of smoothed trajectories shown in blue dashed line in Fig. 3B).

Next, common patterns of gene expression trajectories were identified through trajectory clustering. Conventional clustering methods, such as K-means, K-medoids, and hierarchical clustering, failed to effectively define clusters with distinct trajectory patterns (Fig.S7). Thus, we classified the trajectories based on changes in their slope across different time windows (see Methods). Applying this approach to smoothed trajectories of the top 5000 HVGs yielded six clusters each showing a distinct gene expression pattern over the pseudotime (contour density maps and the average time trajectories in red lines, as shown in Fig. 3C). These patterns include trajectories with monotonic increases or decreases, those with a single turning point, and those with two tuning points. Trajectories with more than two tuning points (15.5%) often correspond to noisy gene expression profiles were therefore excluded from further analysis.

The characterized time trajectory patterns not only provide insights into the gene expression dynamics during the sequential state transitions in iPSC differentiation but also help to identify potential core regulators of these transitions. Here, we hypothesized that genes in a same trajectory cluster is over-represented by the target genes regulated by a same TF. The core TFs were identified by applying the Fisher’s exact test, where genes within each trajectory cluster were compared with the TF target gene sets from the literature-based NetAct database^46^. From this gene enrichment analysis, we identified 16 core TFs enriched in at least one trajectory cluster (q-value < 0.1, as shown in Fig.3D). To reduce false positive TFs resulting from shared target genes, we identified TF pairs with highly overlapping targets (q-value < 10^−20^), and for each pair, we removed the one with the less representative gene expression trajectory (see Methods for details). Under this criterion, JUN and TCF7L2 were removed.

The identified TFs include several key stemness drivers, such as SOX2, POU5F1, KLF8, and MYCN, and TFs involved in epithelial-mesenchymal transition (EMT), including ZEB1, LEF1, and TWIST2. The presence of these core TFs suggests an important role of EMT in the differentiation of iPSCs to DEs and tight coupling between stemness and EMT regulators. Interesting, Scheibner et al. also found that SNAI1 couples the loss of stemness with a partial EMT and maintenance of epithelia traits during definitive endoderm formation^47^. However, the exact interplays of the stemness and EMT regulators during iPSC differentiation are largely undefined. Thus, a systems-biology study on the core GRN of stemness and EMT will provide new insights into the regulatory mechanisms underlying the iPSC-to-DE cell differentiation.

**Fig.4.**
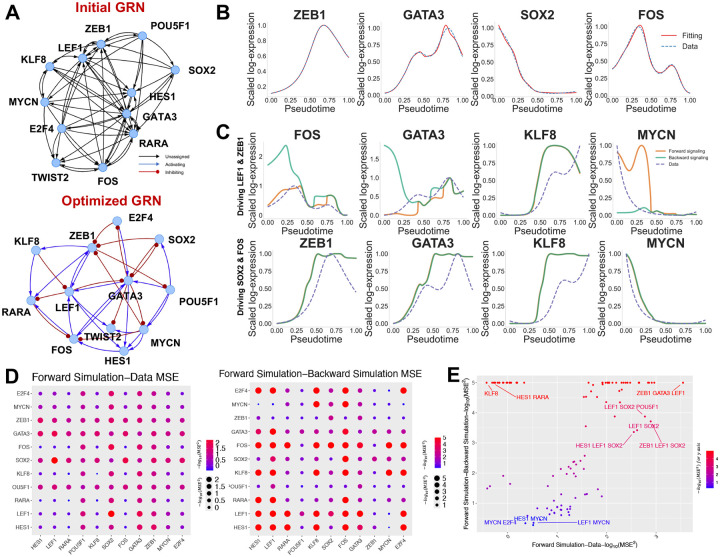
NetDes optimizes a GRN of core TFs driving iPSC differentiation. **(A)** Illustration of network diagrams showing the initial GRN generated using the Rcistarget and TRRUST databases (top) and the optimized GRN inferred by NetDes (bottom). In the initial GRN, black lines and arrows represent regulatory edges with unassigned activation or inhibition. In the optimal GRN, the blue lines and arrows indicate activating edges, while the red lines ending in dots represent inhibitory edges. **(B)** Comparison of expression trajectories of selected TFs between the transcriptomic data (blue dashed lines) and the simulation from the optimized GRN model (red solid lines). **(C)** Comparison of simulated gene expression trajectories when the optimized GRN was driven by forward (orange lines) and backward (green lines) signaling on either LEF1 & ZEB1 (top panel) or SOX2 and FOS (bottom panel). In both panels **B** and **C**, log-normalized gene expression levels from the data (blue dashed lines) are scaled to a range of 0 to 1, while the simulated expression were scaled accordingly. **(D)** The left diagram shows mean squared errors (MSEs) between the experimental and simulated gene expression trajectories, computed for models driven by the expression trajectories of one (diagonal) or two (off-diagonal) TFs. The right diagram shows MSEs between two versions of simulated gene expression trajectories, computed for models driven by TF(s) along the forward direction and the backward directions, respectively. **(E)** Scatter plot summarizing the ability of TFs in driving network gene expression dynamics. The x-axis shows the MSEs between the experimental and forward-simulated gene expression trajectories, while the y-axis shows the MSEs between the forward-simulated and backward-simulated gene expression trajectories. Each dot represents a case when the GRN is driven by one, two, or three TFs.

Once the core TFs were identified from the above steps, we connected the TFs by regulatory interactions using both Rcistarget^39^ and TRRUST^38^ to form an initial GRN (as shown in Fig.4A). In the initial GRN, the activating and inhibiting nature of each edge has not been determined yet and will be resolved by model optimization in NetDes. Two TFs ZNF382 and DNMT1 were removed because they are disconnected from the GRN. Note that we have so far illustrated our approach to constructing a reasonable initial GRN of core TFs, while other approaches, such as GENIE3^23^, GRNBoost2^48^ and DeepSEM^49^ could also be applied (with certain adjustment to focus exclusively on core TFs) to accomplish this task.

### Optimizing a dynamical model of the core TF GRN driving iPSC cell state transitions

As the next step, we applied NetDes to optimize the initial GRN based on gene expression time trajectories. For each target gene (also a TF), we optimized an ODE model by using the gene expression trajectories of the target and its candidate regulators as specified in the initial GRN. After optimizing models for each core TF, we assembled regulatory interactions into an integrated GRN. Depending on the choice of the optimization threshold cutoffs, we build GRNs of different sizes, ranging from 31 to 45 interactions. The best performing GRN contains 36 interactions, as shown in Fig. 4A (see the next section for details on its selection). For most TFs, this approach successfully optimized the ODE models that generates comparable trajectories to those from the observed data (Fig. 4B and Fig. S8). A few TFs, such as KLF8 and E2F4, showed mismatches between fitted and observed trajectories, possibly due to the complexity of their expression dynamics or missing upstream inputs. Overall, the ODE-based optimization accurately reproduces individual gene’s expression even after removing 26 interactions from the initial GRN.

After optimizing the ODE for each TF, we assembled the full set of ODEs for the entire core TF GRN to evaluate whether the gene expression dynamics of the whole system could still be captured by the model. This evaluation is nontrivial because complex regulatory interactions, such as coupled feedback and feedforward motifs, can substantially influence the overall dynamics. Moreover, because iPSC differentiation was experimentally induced by a cocktail of factors^50^ that directly regulate TFs, such as EGR1 and members of the SOX and SMAD families, we simulated the GRN dynamics by driving the system with one, two, or three TFs using their expression trajectories to mimic extrinsic signaling inputs. These simulations allow us to evaluate the extent to which TF expression trajectories within the GRN can be driven by extrinsic signaling and to identify which TFs are likely to drive iPSC differentiation.

In these simulations, we first selected all possible combinations of one or two TFs. For each combination, we simulated the expression trajectories of the remaining TFs by driving the selected TFs according to their trajectories along the pseudotime to induce cell differentiation (referred to as forward signaling). Additionally, we simulated the reverse process by driving the selected TFs with their trajectories along the reverse pseudotime (referred to as backward signaling). Fig. 4C and Figs.S9, S10 show the simulated gene expression trajectories for both forward (orange lines) and backward (green lines) signaling, compared to the observed trajectories from the data (blue dotted lines), for two representative signaling combinations: one driven by LEF1 and ZEB1, and the other by SOX2 and FOS. For LEF1 and ZEB1 (top row in Fig.4C and Fig.S9), several TFs displayed distinct trajectories between forward and backward signaling, indicating the presence of hysteresis, a characteristic feature of multistable dynamical systems. As a result, the simulated gene expression trajectories tended to deviate from the observed data, suggesting that the GRN may encounter barriers in the regulatory landscape when driven by these TFs. We also found that in some TFs, the simulated trajectories exhibited reduced fold changes in gene expression compared to the data, likely due to attenuation through multiple regulatory steps encoded in the coupled ODEs; however, the overall shape of the trajectories was often preserved after appropriate scaling of the observed data. In contrast, for the combination of SOX2 and FOS (bottom row in Fig.4C and Fig.S10), the simulated trajectories from forward and backward signaling closely matched, indicating the absence of hysteresis. In this specific case (although not necessarily for all non-hysteretic combination), the simulated trajectories aligned more closely with the observed data. This suggests that driving the GRN with SOX2 and FOS may create a smoother path in the regulatory landscape for inducing cell state transitions.

To systematically evaluate all combinations of one or two TFs, we plotted heatmaps of the mean square deviations (MSDs) between forward-simulation trajectories and the data (left panel in Fig.4D), as well as the MSDs between forward and backward simulations (right panel in Fig.4D). Fig.4E summarizes both metrics in a scatter plot with annotations on representative TF combinations and selected three top-performed TF combinations. Simulations revealed that single-TF drivers often led to hysteresis and were generally insufficient to fully recapitulate the observed gene expression dynamics, though stemness-associated TFs like SOX2 and MYCN performed better than others. In contrast, dual-TF combinations, particularly those involving ZEB1, SOX2, or GATA3, more effectively drove the GRN without hysteresis. Remarkably, GATA3 was shown to be a driver in the embryonic stem cell differentiation^51^. When used to drive the GRN alone, GATA3 generated favorable results. Furthermore, combining GATA3 with EMT genes such as LEF1, KLF8 and ZEB1 improved the driving performance (e.g., Fig.S11). However, combining GATA3 with other genes did not yield significantly improvement. Furthermore, certain TFs in the system, such as FOS, TWIST2, RARA and E2F4, cannot effectively drive the cell state transitions. Together, the driving simulations show that the optimized GRN can be externally driven to capture the desired cell state transitions, while also identifying candidate drivers for the system.

### NetDes outperformed existing methods in inferring GRNs driving iPSC differentiation

Next, we evaluated the performances of several GRN inference methods in reconstructing regulatory networks capable of recapitulating gene expression states during iPSC differentiation, using both GRN simulation and literature-based evidence. NetDes was benchmarked against five other methods: GENIE3^23^, ppcor^20^, SCODE^26^, SINCERITIES^21^, and CellOracle^27^. For each method, GRNs of the identified core TFs were inferred with varying number of regulatory interactions, ranging from 31 to 45. This selection ensures the constructed GRNs are likely to be connected and not too defensed. We also test two variations of NetDes: one using linear ODE models for optimization (denoted as *NetDes_linear*) and another in which optimization started from a fully connected TF network (denoted as *NetDes_all*). For each inferred GRN, we applied RACIPE (using sRACIPE^52^ package), which generates gene expression profiles by simulating ensembles of ODE models based on the same network topology but with randomly sampled kinetic parameters. Previous studies have shown that RACIPE captures network states that are robust to parameter variations^52–55^, which may mimic cell-to-cell variability and gene expression noise, and that simulated gene expression profiles can be effectively compared with experimental scRNA-seq^56^. Moreover, mathematical modeling using RACIPE was applied to each GRN to ensure that performance evaluation does not depend on the optimized ODE models in NetDes (with fixed kinetic parameters) and treats all methods equally by using randomized parameters.

**Fig.5:**
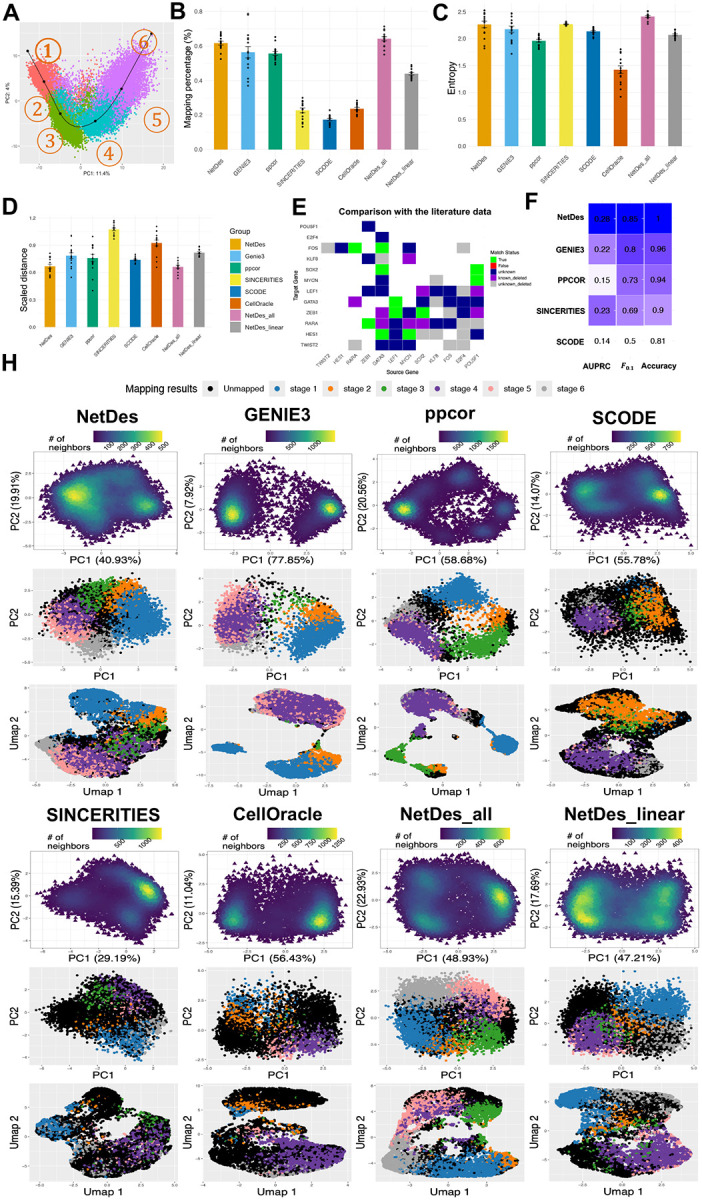
Capability of GRN inference in capturing single cell gene expression states during iPSC differentiation. **(A)** Illustration of six reference points along the principal curve computed by Slingshot using the scRNA-seq data projected onto the first two principal components. Panels **B-D** shows the benchmarking of various methods in inferring GRNs that robustly captures observed gene expression states along iPSC differentiations. For each inferred GRN containing the specified core TFs and network edges ranging from 31 to 45, a population of single cell gene expression data was simulated by RACIPE and was then compared with the reference gene expression profiles. **(B)** Bar plots with scatter points showing the mapping percentages of simulated gene expression profiles to the reference profiles for each inference method. In this comparison, NetDes_all denotes a variation of NetDes where optimizations begins from a fully connected network; NetDes_linear refers to a variation of NetDes which uses a linear ODE model for optimization. Tests were performed on 13 GRNs generated by NetDes, 14 by NetDes_linear, and 15 by the other methods. **(C)** Bar plots with scatter points showing the entropy metric that quantifies the disproportion of mapped states. **(D)** Bar plots with scatter points showing the scaled distances in gene expression of unmapped simulated gene expression profiles to the nearest reference profile. **(E)** Comparison of regulatory interactions between the NetDes-inferred GRN (with the best mapping percentage) and the literature data. Each cell in the matrix represents a regulatory interaction from a regulator (source in columns) to a target (target in rows). Different colors represent interactions (1) not present in the initial GRN (blank), (2) predicted and with literature support (green), (3) with literature support but predicted with wrong activation/inhibition nature (red), (4) predicted but without literature evidence (dark blue), (5) with literature support but removed by NetDes (purple), (6) not predicted and no literature evidence (gray). **(F)** Performance of GRN inference methods in predicting literature-based regulatory interactions. Interactions inferred by a method was filtered to retain only interactions from the initial GRN before performance evaluation. The heatmap and numbers indicate the AUPRC, accuracy, and F_0.1_. **(H)** Simulated gene expression profiles for the inferred GRNs with the highest mapping percentage for each method. Each set of subplots shows the density plot of simulator gene expression projected onto the first two principal components (top), the scatter plot of gene expression colored by their mapped reference states on the PCA space (middle), and the same scatter plot but projected onto the first two UMAP dimensions (bottom).

In the case of iPSC differentiation, we mapped simulated gene expression profiles from RACIPE to six representative gene expression snapshots (denoted as the reference cell states), evenly selected along the principal curve of the scRNA-seq data (Fig.5A), to evaluate how well each GRN topology captures cell states along the continuous differentiation trajectory. Also, we calculated the entropy of model mappings to assess how evenly each GRN captures the continuous cell state transitions (see Methods for details). NetDes achieved highest mapping percentage and entropy among all tested methods, regardless of whether the optimization starts from a literature-based or fully connected initial GRN (Figs. 5BC, Fig. S12). The mapping percentages were drastically reduced when linear models were used in NetDes, suggesting the need of nonlinear ODEs for better network modeling. Moreover, for simulated gene expression profiles that could not be mapped to any reference cell state, those derived from NetDes-inferred GRNs were overall closer to these reference cell states than those from other methods (Fig. 5D). In contrast, GRNs inferred by other methods often produced clusters of simulated gene expression profiles that significantly deviated from the observed data. These results demonstrate that GRNs inferred by NetDes more effectively capture different cell states during differentiation.

For each method, we chose the GRN with the highest mapping percentages and visualized the densities and mappings of RACIPE-simulated gene expression profiles using PCA and UMAP projections (Fig.5G). Simulated profiles from NetDes-inferred GRNs tended to form more continuous trajectories across different reference states in the low dimensional space. In contrast, GRNs inferred by other methods often produced simulated profiles that formed separated clusters, or exhibited incorrect ordering of mapped states.

To further evaluate the influence of the initial GRN in the benchmarking, we filtered the inferred regulatory interactions for each method based on the same initial GRN. The only exception was CellOracle, which incorporates prior information from its own database. With this adjustment, we observed performance improvements in mapping percentages in certain methods (Fig.S13). NetDes still outperformed all other methods in the benchmark. Overall, RACIPE simulations and state mapping analyses show that NetDes consistently outperforms other methods, regardless of whether it starts from a literature-based initial GRN. Nonetheless, we recommend NetDes optimization starting from an initial GRN containing substantially fewer edges, to make model inference more realistic and to reduce computational cost.

Lastly, we compared the TF GRNs inferred by various methods against literature evidence (Supplementary Table I). We found NetDes outperformed all other methods when evaluating the AUPRC, accuracy, and $F_{0.1}$ scores (Figs. 5EF and Figs.S14, S15). Together, these results indicate NetDes as the top-performer in constructing robust and accurate GRN models capturing multi-step cell state transitions.

### Coarse-graining GRN identifies functional circuit motifs

So far, we have applied NetDes to construct a core TF GRN driving iPSC differentiation (Fig. 4A, with the highest mapping percentage). The GRN reveals detailed TF interactions, but how they drive differentiation together remains vague due to the complexity of the GRN. We applied Sacograci^36^ to coarse-grain the GRN into a small circuit motif where nodes represent TF groups and edges represent the collective effects of gene regulation among gene groups.

**Fig.6:**
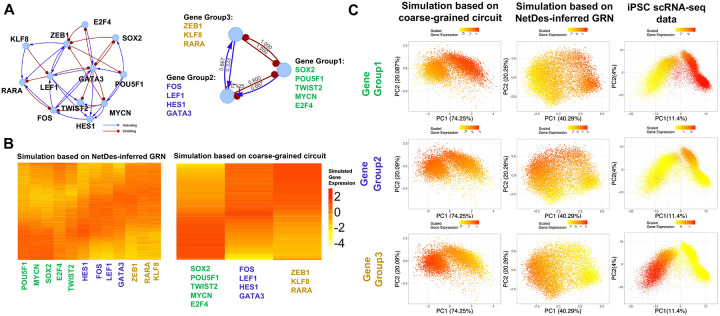
GRN coarse-graining to identify regulatory motif. **(A)** Illustration of the NetDes-inferred full GRN and the corresponding coarse-grained (CG) circuit diagrams inferred by SacoGraci. In the CG circuit, the TFs from the full GRN are grouped into three CG nodes based on expression similarity: Group 1 (SOX2, POU5F1, TWIST2, MYCN, E2F4), Group 2 (FOS, LEF1, HES1, GATA3), and Group 3 (ZEB1, KLF8, RARA). Numbers along each regulatory edge in the CG circuit represents the ratio of activating/inhibitory regulatory interactions present in the full GRN. **(B)** Heatmaps of simulated gene expression for the NetDes-inferred GRN (left) and CG circuit (right). Rows are ordered by inferred pseudotime from Slingshot using the first two principal components of the simulated gene expression profiles. **(C)** Comparison of average gene expression levels for each CG group for the simulated gene expressions from the CG circuit (leftmost column), the simulated gene expression from the full GRN (middle column), and the experimental scRNA-seq data (rightmost column). Each row shows scatter plots of gene expression projected onto its first two principal components, where colors represent mean expression levels for genes of each group. Columns represent simulations from the CG circuit (leftmost), simulations from the TF GRN (middle), and experimental iPSC scRNA-seq data using the top 500 highly variable genes (rightmost).

According to 10000 simulated gene expression profiles of the full GRN using RACIPE (left panel in Fig.6C), we clustered TFs into three groups: (1) SOX2, POU5F1, MYCN, TWIST2, and E2F4, as stemness genes with early expression; (2) FOS, LEF1, and HES, as genes expressed during the intermediate states; (3) ZEB1, KLF8, RARA and GATA3, as genes associated with EMT with late expression. Also, we set the cluster of models into three based on the RACIPE simulated profiles. With the gene and model grouping, we performed 30 iterations of SacoGraci to construct the best coarse-grained (CG) circuit (right panel of Fig.6A) according to the consistency of simulated gene expression profiles of the CG circuit and the full GRN. We found the network interactions from the CG circuit and the full GRN were highly consistent, as shown in right panel in Fig.6A. Furthermore, we found the simulated gene expression distributions for the full GRN (heatmap in the left panel of Fig.6C), those of the CG circuit (heatmap in the right panel of Fig.6C), and the profiles from the experimental scRNA-seq data (all density maps shown in Fig.6B) are qualitatively similar, suggesting a successful coarse-graining of the CG circuit. Interestingly, the optimal CG circuit reveals mutual inhibition between the stemness TFs (Group 1) and EMT TFs (Group 3). In addition, intermediate TFs (Group 2) form negative feedback with the stemness TFs, but positive feedback with the EMT TFs. During iPSC-to-DE differentiation, stemness TFs (Group 1) first activate intermediate TFs (Group 2). As Group 2 expression increases, these intermediate TFs in turn induce EMT TFs (Group 3). Groups 2 and 3 then engage in mutual positive feedback while gradually repressing stemeness TFs (Group 1). Such repression in turn reduces the activities of the Group 2 genes in the later stage through the positive regulation from stemness genes. In summary, GRN coarse-graining uncovers the potential regulatory logic governing multi-step iPSC differentiation.

## Discussion

In this study, we developed a computational method, NetDes, that infers core transcription factor (TF) regulatory networks driving continuous, multi-step cell state transitions using single cell gene expression trajectories. NetDes integrates top-down and bottom-up systems biology by fitting ordinary differential equation (ODE) models to observed gene expression trajectories. The resulting dynamic models are interpretable and allow further simulation and analysis of regulatory systems. We evaluated the performance of NetDes through extensive benchmarking on synthetic gene expression trajectories, a simulated biological gene circuit, and an experimental single-cell RNA-seq dataset, demonstrating its capability to generate robust network models that capture both cellular states and the correct order of cell state transitions.

A major advantage of NetDes is its ability of identify a minimal gene regulatory network (GRNs) of core TFs that drive observed cell state transitions. By explicitly modeling gene expression dynamics using ODEs, NetDes goes beyond static or association-based network inference and enables systems-biology simulation of GRN dynamics under network perturbations or extrinsic signals. These in silico experiments and explorations facilitate the generation of new testable hypotheses for experimental validation, which have the potential to improve causal inference and predictive modeling of regulatory systems. Moreover, simulations for the GRNs driven by combinations of interactions rather than individual edges may help find some synergistic effects overlooked by traditional methods. Compared to existing methods, NetDes utilized a systems-biology dynamical modeling approach that explicitly constructs ODE models to capture the gene expression dynamics during observed multi-step cell state transitions.

We expect NetDes to provide insights into how gene circuits regulate cell state transitions, including developmental progression and cell differentiation.

Despite its advantages, NetDes has several limitations. First, the current method requires high-quality pseudotime or trajectory inference, and their accuracy can affect the performance of network modeling. Second, the model optimization assumes that a deterministic ODE model is sufficient to capture gene expression dynamics, which may perform less well in cases where stochastic dynamics or cell-cell communication are significant. In such scenarios, ensemble-based modeling approaches, such as RACIPE, may better capture these effects. Third, the network optimization step in NetDes is less efficient when a TF has more than ten candidate regulators. This issue can be alleviated through parallelization and the integration of compiled code for optimization. In future studies, NetDes can be extended to model GRNs consisting of different types of gene regulation, such as posttranscriptional regulation. Another direction is to generalize NetDes for analyzing multi-omics datasets, such as transcriptomic, epigenomic, and proteomic data, to enable more comprehensive modeling.

In conclusion, NetDes provides a general approach for mechanistic modeling of transcriptional regulatory networks driving cell state transitions. By combining core TF network inference with ODE-based model optimization and simulations, it offers new opportunities to elucidate design principles of cell fate regulation and to develop effective strategies for driving cell state transitions.

## Methods

### Pseudotime inference

The time-series scRNA-seq data for iPS cell differentiation, provided in the format of counts per million (CPM), were processed by Sctransform^57^ for normalization. The normalized data was then *log*_2_ -transformed (*log*_2_(normalized_data + 1)) and standardized (referred to as the processed data below). We conducted principal component analysis (PCA) on the top 500 biological variable genes (obtained by Scran^58^ using the CPM data) from the processed data and subsequently inferred pseudotime by Slingshot^45^ using the first two principal components. Pseudotime inference allows to characterize gene expression trajectories for NetDes to construct and optimize GRN dynamic models.

### Inference of smoothed gene expression time trajectories

Using the pseudotime value and single cell gene expression for each cell, we applied PseudotimeDE^37^ to infer a smoothed time trajectory of gene expression for each of the top 5000 highly variable genes (HVGs) identified by Scran. Here, PseudotimeDE assumes that each gene in each cell follows a negative binomial distribution and average gene expression along pseudotime is fitted by a spline curve with four knots. Each smoothed gene expression trajectory was then standardized to have zero mean and unit variance.

### Trajectory based gene clustering

These top 5000 HVGs were clustered according to the smoothed gene expression time dynamics. We devised a new approach for clustering because of low performance of several existing algorithms, such as K-means, K-medoids and Hierarchical clustering, in clustering time trajectories (Fig. S7). First, we excluded genes whose processed gene expression data had a standard deviation below a threshold level (0.25). Second, for the rest of the genes, we classified time trajectories according to the increasing and decreasing patterns of gene expression dynamics. To achieve this, for each smoothed time trajectory, we first determined all possible turning points to partition the trajectory into several small segments. For any segment with maximum gene expression changes smaller than 0.2, we excluded the turning points of the segment (*i.e*., one turning point for the first or the last segment, and two turning point points for the rest). We can exclude these turning points here because the proceeding and following segments should have the same increasing and decreasing trends. Finally, we categorized each trajectory into gene clusters according to the increasing and decreasing trends of the curve segments delimited by the remaining turning points, as illustrated in Fig. S7.

To obtain key transcription factors (TFs) driving these gene expression dynamics, we hypothesized that genes with similar gene expression trajectories are likely regulated by common TFs. Thus, we performed enrichment analysis on each gene cluster using a literature-based TF-target gene set database, derived from NetAct^46^, which compiles databases from TRRUST^38^, RegNetwork^59^, TFactS^60^ and TRED^61^. Here, Fisher’s exact test was performed to evaluate the overlapping between genes in a cluster and known target genes for a TF, where the background consisted of all 11231 expressed genes in the scRNA-seq data. Finally, the TFs with adjusted p-value less than 0.1 were selected for building GRNs. To avoid finding false positive TFs due to excessive overlap in target genes, we tried to find TF pairs when overlap occurred between the targets of two TFs (p-value < 10^−20^ in the fisher exact test between the target gene sets in NetAct). Specifically, we retained LEF1 over TCF7L2 as LEF1 showed higher variance in our dataset, whereas TCF7L2 is primarily associated with later gut lineage functions^62^. Similarly, between the AP-1 component FOS and JUN, we selected FOS, which displayed greater expression variability during iPSC differentiation^63,64^.

### Construction of an initial TF regulatory network

Once enriched TFs were identified, we constructed an initial TF regulatory network by connecting the TFs according to the regulatory interactions presented in both the literature-based TF-target databased from TRRUST^38^ and the RcisTarget^39^ database that is derived from cis-regulatory motif prediction. Here, we kept TF-to-TF edges from RcisTarget whose Normailized Enrichment Scores (NES) are above one to include more potential edges in the initial network, with the expectation that unrelated edges will be removed during GRN optimization.

### Optimizing a nonlinear dynamic model of GRN

For each TF in the initial GRN, we optimized a nonlinear ODE model according to the smoothed gene expression time trajectories of itself and its TF regulators (Fig.S8). To ensure uniformity in trajectory fitting and reduce the effects of cell distribution variability along pseudotime, we selected M=201 evenly spaced time points along each trajectory. Additionally, we scaled the gene expression values along each trajectory by their maximum value.

The ODEs of the dynamic model were adopted from a common approach in systems-biology modeling of gene regulatory circuits/networks.

(1) $$\frac{dx_{i}}{dt}=g_{i}\prod_{j}\left\{\lambda_{ij}+\frac{\left(1-\lambda_{ij}\right)}{\left[1+{\left(\frac{y_{j}}{R_{ij}}\right)}^{n_{ij}}\right]}\right\}-k_{i}x_{i}$$

where $x_{i}$ represents the expression levels of target gene $i,y_{j}$ represents the expression levels of regulator $j;g_{i}$ and $k_{i}$ are the basal production rate and degradation rate for gene i; the product is over all regulators j of gene $i;\lambda_{ij},R_{ij}$ and $n_{ij}$ are the maximal fold change, Hill threshold, and Hill coefficient for the regulation from regulator j to gene i^54^. Gene j activates i when $\lambda_{ij}>1$, and inhibits i when $\lambda_{ij}<1$.

Under the quasi-steady state assumption during the dynamic progress, Equation (1) leads to

(2) $${\text{log}}_{2}\left(x_{i,l}\right)=\sum_{j}{\text{log}}_{2}\left(\lambda_{ij}+\left(1-\lambda_{ij}\right)\frac{1}{1+{\left(\frac{y_{j,l}}{R_{ij}}\right)}^{n_{ij}}}\right)+C$$

where $C={\text{log}}_{2}\left(\frac{g_{i}}{k_{i}}\right)$. The additional subscription l represents the indices of the data points along the time trajectory. To allow to trim unnecessary interactions, we performed nonlinear fitting, for each gene i, with a regulation term:

(3) $$\text{argmin}\left\{\frac{1}{M}\sum_{l=1}^{M}{\left({\text{log}}_{2}\left(x_{i,l}\right)-y_{i,l}\right)}^{2}+w_{i}\sum_{j}{\left(\lambda_{ij}-1\right)}^{2}\right\}$$

In the fitting, ${\text{log}}_{2}\left(x_{i,l}\right)$ is computed by using Equation (2), $y_{i,l}$ for each gene is obtained from the smoothed gene expression trajectories, and $w_{i}$ is the tuning parameter for the regulation term. The summation is over all M=201 data points. When the optimized $\lambda_{ij}$ approaches to one, the interaction from genes j to i is essentially removed from the dynamic model. But instead of directly removing interactions by a LASSO process, we performed multiple rounds of model fitting (as described in the following sections) by considering all possible subsets of regulators, from which we identified a more robust prediction of regulators.

### Initial model optimization (first stage)

To ensure robust model fitting and prevent the nonlinear fitting from being trapped in local minima, we devised an approach consisting of four stages for model optimization (Fig.S1A). During the first stage, we performed an initial round of optimization without regulation. First, a substantial number of initial parameters were sampled for the optimization as follows. For each gene i and its regulator j, we sampled the initial $\lambda_{ij}$ from $4>\lambda_{ij}>2$ (for activation) and $0.5>\lambda_{ij}>0.25$ (for inhibition) to ensure all activation/inhibition combinations from all regulators of gene i. In addition, we uniformly sampled the initial $R_{ij}$ from $\left(0.3\cdot \text{max}\left(y_{j}\right),0.7\cdot \text{max}\left(y_{j}\right)\right.$), the initial $n_{ij}$ from (0.01, 0.99), and the initial C from (−2, 2). This approach results in a total of $2^{N_{i}}$ sets of initial ODE parameters, where $N_{i}$ is the number of regulators of gene i. Second, starting from each of the $2^{N_{i}}$ sets of initial parameters, we applied five-fold cross-validation (CV) to fit the model using Eq. (3) but without the regulation terms $\left(w_{i}=0\right)$ (Fig.S1A, step 1). For the five-fold CV, we separated the data evenly into five parts based on the pseudotime and, for each iteration, used four parts to fit the model and the rest one as test data to calculate the mean squared errors (MSEs). After the five-fold CV, we computed the mean MSE as the final MSE under this parameter set. Third, among all the fitted models, we selected the models with the best 50% (if $N_{i}\leq 6$) or the top 50 (if $N_{i}>6$) models in MSEs, and then we re-optimized these models starting from resampled initial parameters (but keeping the same $\lambda_{ij}$ ranges) again for ten times to further improve the fitting outcomes (Fig.S1A, step 2). Finally, the top 100 models in MSEs (or all top 50% models if the total number is less than 200) will be used later for a second stage of model optimization where regulation terms were added (described in the next section).

### Tuning parameter estimation and optimization with regulation (second stage)

Right after the first stage of model optimization without using the regulation terms, we estimated the tuning parameters so that the regulation terms on the maximum fold change parameters could be properly incorporated during the second stage (Fig.S1A, step 3). For each gene i, we selected the initial parameters from the top four fitted models according to the MSE values (see the 1^st^ stage). We selected a series of tuning parameters $w_{i}$ (a total of 20 numbers by default) by evening sampling $\text{ln}\left(w_{i}\right)$ from −15 to 2. For each tuning parameter, we performed more model fitting using Equation (3) and with the regulation term by a five-fold CV test. From the plot of $w_{i}$ (x-axis) *v.s*. log (*mean* (*MSE*)) (y-axis, the average is over the above-mentioned four fitting cases), we fitted a spline curve and determined the tuning parameter $w_{i}$ by the minimum of the spline curve (Fig.S1C). This procedure was repeated to determine the tuning parameter for every gene.

From the outcomes of the 1st stage model optimization, we then performed a second stage optimization by incorporating the regulation terms as follows (Fig.S1A, step 4). The top 100 models in MSEs (or top 50% models if the total number of models is less than 200) were optimized again with the regulation terms. Here, for each model, its initial parameters were re-sampled while keeping the same patterns of activating/inhibiting edges (*i.e*., $\lambda_{ij}$ were re-sampled according to the same ranges as specified in the 1^st^ stage). After this round of optimization, the optimized models were ranked again according to the MSEs. Incorporating regulation for the fold change parameters alleviates potential overfitting by effectively reducing the number of regulators for each gene.

### Optimization on reduced regulatory scenarios (third stage)

To further improve the robustness of optimization and determine a minimal regulatory model recapitulating gene expression trajectory, we employed a third stage optimization on models where subsets of regulators were selected for additional optimization but without regulation. First, from the optimized models in the previous step (step 4), we identified all removable incoming edges for each gene i (Fig.S1A, step 5). Here, we considered an edge to be *removable*, if the optimized $\lambda_{ij}$ can be both $\lambda_{ij}>1$ and $\lambda_{ij}<1$ within all the optimized models by step 4. Second, we generated all possible regulatory scenarios for gene i, where a subset of removable incoming edges were deleted. For each of such scenarios, we fitted the ODE model again ten times, where the new optimization starts by using the fitted parameters from the top 10 (instead of top 100 to reduce computational cost) optimized models from step 4. In this fitting step, we did not use regulation terms and cross validation. Newly fitted models and their corresponding regulatory scenarios were ranked by a reducibility index I, defined by

(4) $$I=\sum_{h=1}^{10}\frac{MSE_{h}}{MSE_{\text{ori,h}}}$$,

where $MSE_{h}$ are the optimized MSE under different initial parameters, and $MSE_{\text{ori,h}}$ is the original MSE from the $h^{\text{th}}$ model in the top 10 model optimization in step 4.

The reducibility index highlights the impact of edge deletions on model fitting. For the regulatory scenarios whose reducibility indices are below a specific cutoff value, we determined the optimal regulatory scenario according to the fewest number of edges. If there are multiple of such scenarios, the optimal scenario was selected to be the one with the lowest reducibility index. By selecting a specific cutoff value, we obtained optimal regulatory scenarios for each gene and built a GRN by assembling regulatory interactions from all genes. By choosing different cutoff values, we can generate GRNs of varying sizes.

### Optimization to refine and finalize the ODE model (final stage)

Once we constructed a GRN corresponding to a specified reduced regulatory scenario, we finalized a corresponding ODE mode that specifies the activation/inhibition nature of each interaction using the final optimization steps (Fig.S1B, step 8). First, an initial optimization was performed without regulation for all optimized models in step 4 but under the reduced regulatory scenario (Fig.S1B step 9, like Fig.S1A step 1). Here, the initial parameters of the reduced models were derived from the corresponding fitted parameters of these full models. Second, the top four fitted models from the previous step was used to estimate the tuning parameters (Fig.S1B step 10, similar to Fig.S1A step 3). Third, another round of optimization was then performed for all models in step 9 but with regulation terms (Fig.S1B step 11, similar to Fig.S1A step 4). Finally, we identified a subset of the optimized models with the most frequent activation/inhibition pattern of the regulators according to the signs of $\lambda_{ij}$. From these models, we identified the best model according to the smallest MSE (Fig.S1B step 12). This step generated the final ODE model of gene i. The process was repeated for each gene to complete the dynamical model of the whole GRN.

### ODE Simulations of cell state transitions driven by extrinsic signals

After the GRNs and the associated ODEs were obtained from model optimization, the gene expression dynamics of the GRNs can be simulated by integrating the ODEs with the 4th Order Runge-Kutta Method. In the simulations, we started from an initial condition where gene expression levels were selected from the initial time point of the smoothed time trajectories and simulated the ODE model until it reaches to a stable steady state. This stable steady state was used as the initial condition for further simulations. To evaluate the dynamical response of the GRN to extrinsic signals, we performed two simulations on the dynamic model by driving the system with the observed gene expression trajectories on one, two or three TFs in the GRN. In the first simulation, the system starts from the initial state and is driven by the gene expression trajectory of the TF (or TFs) along the forward time direction. In the second simulation, the system starts from the final state of the first simulation and is driven by the gene expression trajectory of the TF (or TFs) along the backward time direction. These two simulations allow us to evaluate how effectively the whole system is driven by extrinsic signals on selected TF(s) to allow cell state transitions.

### Mapping GRN gene expression states to single cell RNA-seq data

We performed additional model simulations of the inferred GRNs and checking how consistent the GRN gene expression profiles are with the gene expression states from the single cell RNA-seq data. To do this, we selected six time points evenly along the smoothed time trajectories of the GRN TFs as the reference states. Choosing six time points allows for a more comprehensive representation of the dynamic changes in gene expression over time, capturing key cell state transitions and ensuring a robust comparison between the model simulations and the observed gene expression states from the scRNA-seq data. The mapping procedure consists of the following steps.

First, we applied, a mathematical modeling method, Random Circuit Perturbation (RACIPE)^52–55^, on each inferred GRN, to simulate single cell gene expression profiles. Here, according to the topology of the GRN, RACIPE generates an ensemble of ODE models with randomly selected kinetic parameters and obtained a stable steady state for each model via ODE simulations. Ensembled-based mathematical modeling would allow to capture cell-to-cell variability in a single-cell population and the effects of GRN regulation. For each inferred GRN, RACIPE was applied to generate 10,000 gene expression profiles of the GRN genes.

Second, simulated gene expression data were log-normalized so that each gene’s distribution matches that of the smoothed time trajectory. Specifically, the gene expression $u_{i}$ of gene i in a RACIPE model was log transformed by ${log}_{2}\left(u_{i}+k_{i}\right)$, where $k_{i}$ is a gene specific constant for the GRN simulation. Afterwards, standardization was applied to the log transformed data. This treatment helps to make RACIPE-simulated data more closely with the experimental expression data. For each gene i, we selected $k_{i}$ such that ${log}_{2}\left(k_{i}\right)$ was evenly sampled between −10 and 5. We then identified the optimal $k_{i}$ that maximized the overlap between the distribution of the log-normalized RACIPE models and that of log-normalized gene expression from 201 evenly distributed points along the smoothed time trajectory.

Third, to establish the mapping, we calculated the Euclidean distance in high-dimensional gene expression profiles $D_{a,r}$, which is between a RACIPE model a and one of the six reference states r (after log-normalization). The RACIPE model a was successfully mapped to the reference state r, when

(5), $$D_{a,r}\leq D_{r}$$

where $D_{r}$ is the cutoff distance for the reference state r, corresponding to the bottom 5% of Euclidean distances between a random gene expression profile and the gene expression profile from the reference state r. The random gene expression profile was generated from a normal distribution with the same mean and standard deviation of RACIPE simulated gene expression profiles. If the model a can be mapped to more than one reference states, the model is mapped to the reference state with the minimum $D_{a,r}/D_{r}$.

We quantified how well RACIPE models are mapped to any of the six reference states by a metric called *weighted mapping percentage*. Here, the mapping of each RACIPE model is weighted by the inverse of its local density of gene expression from all 10,000 RACIPE models. The local density of a model a is defined as

(6), $$\rho_{a}=15/\pi R_{a}^{v}$$

where $R_{a}$ is Euclidean distance of the gene expression profiles, considering all data dimension v, between the model and the 15^th^ nearest neighbors. In our application, we also calculated the low-dimensional local density, which considered the $R_{a_{-}\text{low}}$ as the distance within the first three principal components (PCs) obtained from PCA, where the dimension v replaced by $v_{\text{low}}=3$.

Then, the weighted mapping percentage is defined as

(7), $$p_{tot}=\sum_{a}\frac{\sum_{a\in A_{r}}\frac{1}{\rho_{a}}}{\sum_{a\epsilon A}\frac{1}{\rho_{a}}}$$

where $A_{r}$ presents all the RACIPE models mapped to the reference state r, and A presents all the RACIPE models. Here, weights $\rho_{a}$ inversely proportional to model density were used to avoid favoring top-ranked GRNs that achieve high mapping percentages merely by aligning well with only one or two reference states. We incorporated both low-dimensional local density (using the first three principal components) and full-dimensional density when calculate the weighted mapping percentages. It is worth noting that local density computed in the full-dimensional space tends to produce highly skewed distributions. Hence, our analysis mainly based on low-dimensional mapping results.

To evaluate a GRN’s overall performance of capturing various states along a continuous state transition, we defined GRN entropy as

(8), $$H=-\sum_{i=1}^{n_{r}}p\left(r_{i}\right){\text{log}}_{2}p\left(r_{i}\right)$$

where $p\left(r_{i}\right)$ represents the percentage of simulated RACIPE models that mapped to a reference state $r_{i}$, and $n_{r}$ is the number of the reference states. Higher entropy indicates that a GRN captures states more evenly, whereas lower entropy suggests the GRN is biased toward specific states.

We also used another metric to qualify model deviations from the reference gene expression. For those RACIPE models that are not mapped to any reference state, we evaluated how each model deviates from the closest reference state in gene expression. Thus, we computed the average normalized distance

(9), $$d_{u}=\frac{1}{N_{u}}\sum_{b=1}^{N_{u}}\text{min}\left(\frac{D_{b,r}}{D_{r}}\right)$$

where the summation is over any unmapped RACIPE models $b,D_{b,r}$ is the Euclidean distance in high-dimensional gene expression profiles between unmapped RACIPE model b to a reference state $r.N_{u}$ is the total number of unassigned RACIPE models. Similarly, we also computed the average normalized distance $d_{\theta }$ for random profiles, where the RACIPE simulated gene expression profiles are replaced by the random gene expression profiles previously generated from normal distributions. Finally, we defined the model deviation metric as $\omega =\frac{d_{u}-1}{d_{\theta }-1}$. A high model deviation metric typically suggests the existence of unmapped gene expression cluster(s) from GRN simulations, in which case the GRN is incapable of fully recapitulating observed gene expression states.

### Simulation of a gene circuit driving ICM-to-TE transition

To simulate the gene expression dynamics of the gene circuit driving ICM-to-TE transition, we set the expression trajectory of the signaling gene $\text{Fgf}\left(x_{4}\right)$ as an input signal changing from its initial value of 150 (arbitrary unit) to its final value of 25 over the course of the simulation.

(10) $$x_{4}(t)=\left\{\begin{array}{cc} 150-125*\frac{1-\text{cos}\left(\frac{\pi \text{t}}{\text{T}/2}\right)}{2}, & 0\leq \text{t}\leq \text{T}/2\\ 25, & \text{T}/2<\text{t}\leq \text{T} \end{array},\right.$$

where T is the total simulation time, which is set to 1000.

For other three genes $\text{Oct}4\;\left(x_{1}\right),\text{Cdx}2\;\left(x_{2}\right)$ and $\text{Esrrb}\;\left(x_{3}\right)$, we simulated their gene expression dynamics according to:

(11), $$\frac{dx_{i}}{dt}=g_{i}\prod_{j\neq i}\left\{\lambda_{ij}+\frac{\left(1-\lambda_{ij}\right)}{\left[1+{\left(\frac{x_{j}}{R_{ij}}\right)}^{n_{ij}}\right]}\right\}-k_{i}x_{i}$$

The parameter set for the simulation are shown in Supplementary Table 2.

### Synthetic dataset for benchmarking

To evaluate the performance of network inference, we generated synthetic datasets of time-series gene expressions for regulators and target genes. We randomly generated ten curves from polynomials of either fourth or fifth degrees to represent the gene expression trajectories of ten genes (see Supplementary Equation 1 for the formulae of the curves). Subsequently, we randomly selected m (from 2 to 7) out of the ten genes as the gene expression trajectories of the regulators ($x_{j}$ in the following formula) and simulated the steady-state gene expression of its target gene (y) by the following model with randomly chosen parameters:

(12),, $${\text{log}}_{2}\left(y\right)=\sum_{j}{\text{log}}_{2}\left(\lambda_{j}+\left(1-\lambda_{j}\right)\frac{1}{1+{\left(\frac{x_{j}}{R_{j}}\right)}^{n_{j}}}\right)+C$$

where ${\text{log}}_{2}\left(\lambda_{j}\right)$ randomly picked uniformly from either (−3, −1) for inhibition or (1,3) for activation, $R_{j}$ is randomly selected from $\left(0.1\cdot \text{max}\left(x_{j}\right),0.9\cdot \text{max}\left(x_{j}\right)\right),n_{j}$ from (2.5, 4.5) and C from (−2, 2).

For the benchmarking test, all ten time trajectories and the simulated time trajectory of the target gene y were provided as the input, where the ground truth is the membership of the true regulator genes. Additionally, from the false regulators, we randomly selected $m_{d}$ genes (ranging from 1 to 9-m) as the decoy regulators in the test. Thus, each testing case has m true regulators, $m_{d}$ decoys and one target gene. For each gene, we evenly selected 201 time points along the time trajectory and used the corresponding gene expression data as the input data for the tests. In the current benchmark test, we chose $9\geq m+m_{d}\geq 4$, and, for each combination of $\left(m,m_{d}\right)$, we conducted a random sampling of the true regulators and decoys for three times. In total, we generated 81 different test cases for benchmarking.

To test the performance of an algorithm in identifying the true regulators under varying levels of noise, we added Gaussian noise of different intensity to the gene expression of the 201 time points from the original gene expression trajectories of the regulators and targets by

(13), $$G^{\prime }(t)=G(t)+\gamma \cdot N(0,1)\cdot G(t)$$

where G(t) is the deterministic gene expression at time $\text{t},G^{\prime }(t)$ is the noisy gene expression at time t,γ is a scaling factor that adjusts the intensity of the noise. And N(0,1) represents the Gaussian noise with a mean of 0 and a variance of 1. For each noise level, we tested all the 81 combinations of true regulators and decoys. The synthetic benchmark data set is available in our GitHub repository (see Data Availability)

### Implementation of other GRN reconstruction methods

We employed multiple GRN reconstruction methods, GENIE3^23^, ppcor^20^, SCODE^26^ alongside NetDes, to benchmark their performance using both the synthetic data sets and experimental iPSC data. Additionally, we also applied SINCERITIES^21^ and CellOracle^27^ on the iPSC scRNA-seq data to evaluate their performance in inferring TF GRNs and recapitulating dynamical behaviors of cell state transitions. The implementation of these methods is outlined as follows.

The ppcor^20^ method calculates partial and semi-partial correlation coefficients between regulators and target genes. In our testing, we used semi-partial correlation for obtaining the interactions with sign. For synthetic data, we opted for the Kendall method to compute the correlation matrix, instead of using the default Pearson correlation coefficients due to errors associated with zero eigenvalues. For the iPSC scRNA-seq data, we applied ppcor to the processed data, and GRN interactions were ranked based on the absolute value of Pearson (or Kendall) correlation coefficients.

GENIE3^23^ employs a tree-based model to predict the interactions between genes. We utilized the default setting of GENIE3 for its application to both the synthetic data and processed iPSC data. To specify the activation and inhibition nature of each regulator-target regulation, we assigned the sign of Pearson correlation coefficients to the interaction. Additionally, the regulatory edges were ranked by the interaction weights inferred by GENIE3.

SCODE^26^ utilizes a linear ODE method to fit the GRN dynamics. We used default setting and running 100 iterations of optimization for both the synthetic data and processed iPSC data. Regulatory edges were ranked by the absolute value of correlation coefficient defined by SCODE.

CellOracle^27^ involves learning from chromatin accessibility data and gene expression data. We specifically tested this method using the raw iPSC scRNA-seq data. In the implementation, we incorporated the day labels from scRNA-seq data as cluster information. We selected the prebuild promoter based GRN from human data available in CellOracle and combined the interactions inferred from the four gene expression clusters to compile a final interaction list. Regulatory interactions were ranked by the p-value in ascending order.

SINCERITIES^21^ analyzes gene expression changes over time with the Kolmogorov-Smirnov statistic. In the implementation, we chose the ridge regression from four different regularization regression strategies to find the gene interactions with sign using the processed iPSC data. The regulatory edges were ranked based on the absolute values of their weights, as determined by SINCERITIES, which reflects both the strength and direction of interactions. This ranking was used to construct GRNs of different sizes.

### Performance Assessment for benchmarking using the synthetic data

For benchmarking GRN inference using the synthetic data, we evaluated their performance against ground truth by calculating area under the receiver operating characteristic curve (AUROC) and area under the precision-recall curve (AUPRC). For methods capable of predicting the interactions with signs, we also accessed the AUROC and AUPRC for activating and inhibiting interactions separately.

We obtained the ranked interactions as predicted from each method. Each interaction was weighted using log $\left(A–\pi_{e}+2\right)$ in the process of calculating precision and recall, where A is the number of interactions and $\pi_{e}$ is the rank of the interaction e. It is worth noting that NetDes provides a ranking for different combinations of regulators based on reducibility index I, instead of a ranking for individual regulators. Thus, in this benchmark test, to compare with other methods, we instead first identified the best predicted combinations for each possible number of regulators and then ranked regulators based on how frequently they showed up in these combinations. Although this evaluation approach did not fully capture NetDes’s performance, NetDes still outperformed other methods in predicting regulators in the benchmark test incorporating gene expression noise and decoy genes.

### Performance Assessment for iPSC GRN inference using the literature data

NetDes optimizes a GRN model starting from an initial network composed of interactions derived from TF-target databases, including TTRUST (based on text-mining) and Rcistarget (based on TF binding motif data). As a result, the interactions considered by NetDes already have some supporting evidence. However, these interactions may not be direct or context-specific regulation. To further assess the performance of GRN reconstruction on the iPSC dataset, we performed an extensive literature survey to identify TF-target relationships within the initial GRN that are supported by published evidence. Details of these literature-supported interactions are provided in SI Table1, and they were regarded as the ground truth in this benchmarking.

For each GRN inference method, we constructed core TF networks, each containing a different number of regulatory edges, and only included interactions present in the initial GRN for benchmarking. The only exception for this filtering step was CellOracle, which replied on its own pre-built GRN. We considered GRNs with 31 to 45 edges (after filtered based on the initial GRN) to ensure that the inferred GRNs remained connected while being substantially smaller than the initial network (which contains 59 edges). For the benchmarking, we evaluated the AUPRC, accuracy, and $F_{0.1}$ scores according to the literature-based evidence. The AUPRC values were calculated over GRNs with 31–45 edges, whereas F_0.1_ and accuracy correspond to the highest values achieved among the reconstructed GRNs. In this analysis, a true positive is an interaction both identified in the GRN and supported by the literature with consistent interaction sign, a false positive is an interaction agreed upon by both but with inconsistent signs, and a false negative is an interaction verified in the literature but missed by the GRN.

### GRN Coarse-graining

We applied a coarse-graining method, Sampling Coarse-Grained Circuits (SacoGraci)^36^, to construct small gene circuits from the optimal core TF GRN inferred by NetDes. SacoGraci constructed coarse-grained circuits by a Markov Chain Monte Carlo sampling of small gene circuits, whose simulated gene expression states match those from the full GRN. In this study, we manually categorized genes into three groups according to their RACIPE simulated expression patterns along pseudotime (early, intermediate and late activation) (Fig.6B). In addition, simulated gene expression profiles from the full GRN were clustered by using hierarchical clustering analysis (three clusters, distances based on signed Pearson correlation, and Ward.D2 linkage). We used the default parameters for SacoGraci and ran the optimization 30 times to obtain the best coarse-grained circuit according to the scores computed in SacoGraci.

## Supplementary Material

Supplement 1

## Data Availability

NetDes package is available at both GitHub https://github.com/lusystemsbio/NetDes and PyPI https://pypi.org/project/NetDes/. Analysis scripts, including in-silico benchmarking, scRNA-seq application and TF regulatory network modeling, are available at https://github.com/lusystemsbio/NetDesAnalysis/.

## Data availability

The data used in this study are as follows: iPSC scRNA-seq data are available at https://zenodo.org/records/3625024#.Xil-0y2cZ0s; the synthetic benchmark datasets are available at https://github.com/lusystemsbio/NetDesAnalysis/; the RcisTarget human motif database is available at https://resources.aertslab.org/cistarget/databases/homo_sapiens/hg19/refseq_r45/mc9nr/gene_based/; and the NetDes human TF–target database is available at https://github.com/lusystemsbio/NetAct/blob/master/data/hDB.rdata.
