# Supplementary material for: Building dynamical models of multi-step state transitions from single cell gene expression trajectories
Source: bioRxiv. 2025 Dec 11:2025.12.08.693064. Preprint. [Version 1] doi: 10.64898/2025.12.08.693064 (PMC12709465; doi:10.64898/2025.12.08.693064)
Supplement: Supplement 1 [file media-1.pdf]

Building dynamical models of multi-step state transitions from single cell gene expression trajectories

Yukai You[1,2], Cristian Caranica[1,2], and Mingyang Lu[1,2*]

Appendix

Table of Contents

Supplementary Equation 1. Mathematical formulae of synthetic trajectories 3

Supplementary Table 1. Literature evidence for TF-target interactions from the initial GRN. 4

Supplementary Table 2. Model parameters of the optimized ODEs for the gene circuit governing ICM-TE transition. 6

Supplementary Fig 1. Flowchart diagram for the ODE parameter optimization procedure in NetDes. 7

Supplementary Fig 2. Additional model optimization results for the application to iPSC differentiation scRNA-seq data. 8

Supplementary Fig 3. Ten time trajectories for synthetic benchmarking. 9

Supplementary Fig 4. AUROC results for benchmarking NetDes and other inference methods on the synthetic dataset. 10

Supplementary Fig 5. NetDes inference of GRN driving cell state transition from inner cell mass (ICM) to trophectoderm (TE). 11

Supplementary Fig 6. GRN simulations for NetDes inferred GRNs for the ICM-to-TE state transition. 13

Supplementary Fig 7. Comparison of clustering algorithms on gene expression trajectories. .14

Supplementary Fig 8. Comparison between experimental and fitted gene expression trajectories for each TF. 15

Supplementary Fig 9. Comparison of simulated gene expression trajectories when the optimized GRN was driven by both LEF1 & ZEB1. 16

Supplementary Fig 10. Comparison of simulated gene expression trajectories when the optimized GRN was driven by both SOX2 & FOS. 17

Supplementary Fig 11. Comparison of simulated gene expression trajectories when the optimized GRN was driven by LEF1, ZEB1 and GATA3. 18

Supplementary Fig 12. Mapping percentages in full-dimensional expression space without providing initial GRN (related to Fig.5). 19

Supplementary Fig 13. Capability of GRN inference in capturing single cell gene expression states during iPSC differentiation (related to Fig.5). 20

Supplementary Fig 14. Comparison of inferred regulatory edges from each method against literature evidence. 22

Supplementary Fig 15. Comparison of inferred regulatory edges from each method against literature evidence. 23

Supplementary Equation 1. Mathematical formulae of synthetic trajectories

$$I_1(\mathrm{x}) = \frac{1000 + x \cdot (x - a_0) \cdot (x - a_1) \cdot (x - a_2) \cdot (x - a_3)}{100}$$

$$I_2(\mathrm{x}) = \frac{1400 - (x - a_4) \cdot (x - a_5) \cdot (x - a_6) \cdot (x - a_7) \cdot (x - a_8)}{100}$$

$$I_3(\mathrm{x}) = \frac{5500 + (x - a_9) \cdot (x - a_{10}) \cdot (x - a_{11}) \cdot (x - a_{12}) \cdot (x - a_{13})}{100}$$

$$I_4(\mathrm{x}) = \frac{1000 + (x - a_{14}) \cdot (x - a_{15}) \cdot (x - a_{16}) \cdot (x - a_{17}) \cdot (x - a_{18})}{100}$$

$$I_5(\mathrm{x}) = \frac{300 \cdot x - 1.5 \cdot (x - a_{19}) \cdot (x - a_{20}) \cdot (x - a_{21}) \cdot (x - a_{22})}{100}$$

$$I_6(\mathrm{x}) = \frac{270 - (x - a_{23}) \cdot (x - a_{24}) \cdot (x - a_{25}) \cdot (x - a_{26}) \cdot (x - a_{27})}{100}$$

$$I_7(\mathrm{x}) = \frac{4600 + (x - a_{28}) \cdot (x - a_{29}) \cdot (x - a_{30}) \cdot (x - a_{31}) \cdot (x - a_{32})}{100}$$

$$I_8(\mathrm{x}) = \frac{100 + (x - a_{33}) \cdot (x - a_{34}) \cdot (x - a_{35}) \cdot (x - a_{36})}{100}$$

$$I_9(\mathrm{x}) = \frac{3200 - (x - a_{37}) \cdot (x - a_{38}) \cdot (x - a_{39}) \cdot (x - a_{40})}{100}$$

$$I_{10}(\mathrm{x}) = \frac{3000 - 160 \cdot x + (x - a_{41}) \cdot (x - a_{42}) \cdot (x - a_{43}) \cdot (x - a_{44})}{100}$$

Supplementary Table 1. Literature evidence for TF-target interactions from the initial GRN. For every regulatory interaction from the initial GRN, the column named "Interaction type" indicates whether the transcriptional regulation is activating or inhibitory (blank if unknown). The column named "Reference" describes literature evidence and their references. An "NA" in this column represents that no supporting publication was identified.

Source	Target	Interaction type	Reference
E2F4	TWIST2		NA
FOS	TWIST2		NA
LEF1	TWIST2		NA
MYCN	TWIST2		NA
RARA	TWIST2		NA
GATA3	TWIST2		NA
LEF1	HES1		NA
LEF1	RARA		NA
LEF1	ZEB1	Activation	(indirect)The EMT induced by LEF-1 is associated with an increase in the mRNA levels of ZEB1[1]. (https://doi.org/10.1016/j.bbrc.2013.11.031) Zeb1 is indirect recruited to regulatory regions by Lef1 results in gene activation[2] . (https://doi.org/10.15252/embj.201797115)
LEF1	GATA3	Inhibition	LEF-1 suppresses GATA-3 DNA-binding activity and Th2 cytokine production[3]. (https://doi.org/10.1111/j.1365-2567.2008.02854.x)
FOS	RARA		NA
E2F4	GATA3		NA
E2F4	HES1		NA
E2F4	LEF1		NA
E2F4	RARA		NA
E2F4	ZEB1		NA
SOX2	GATA3		NA
SOX2	HES1		NA
SOX2	LEF1	Inhibition	Sox2 acts as a repressor to directly modulate Wnt-responsive transcription of the Lef-1 gene promoter[4]. (https://doi.org/10.1152/ajplung.00157.2013)
SOX2	ZEB1	Inhibition	(indirect) SOX2 and OCT4 transcriptionally regulate miR200, which in turn represses ZEB genes[5]. (https://doi.org/10.1073/pnas.1212769110)
SOX2	RARA	Inhibition	(indirect) SOX2 knockdown in HF2303 GBM Cells increases RARA expression by 2.2 fold change[6]. (https://doi.org/10.1016/j.neo.2014.03.006)
ZEB1	RARA	Activation	(indirect) Knockdown of ZEB1 suppresses the RARA-mediated EMT phenotype[7]. (https://doi:10.1016/j.molonc.2014.09.005)
POU5F1	LEF1	Activation	Oct4 induces EMT through LEF1/β-catenin dependent WNT signaling pathway in hepatocellular carcinoma[8]. (https://doi.org/10.3892/ol.2017.5788)
POU5F1	RARA		NA
POU5F1	GATA3		NA
POU5F1	ZEB1	Inhibition	(indirect) SOX2 and OCT4 transcriptionally regulate miR200, which in turn represses ZEB genes[5]. (https://doi.org/10.1073/pnas.1212769110)
ZEB1	LEF1		NA
GATA3	RARA	Activation	(indirect) RARα can be recruited to GATA binding sites by protein interactions[9]. (https://doi.org/10.1128/MCB.24.15.6824-6836.2004)
FOS	ZEB1		NA

Source	Target	Interaction type	Reference
GATA3	HES1	Activation	Chip-seq shows that GATA3 binds to the promoter of HES1[10]. (https://doi.org/10.1016/j.stem.2020.03.005); HES1 is upregulated following GATA3 overexpression[11] (https://doi.org/10.1038/ncomms11171)
FOS	GATA3		NA
GATA3	LEF1		NA
POU5F1	HES1		NA
FOS	LEF1		NA
KLF8	RARA		NA
RARA	GATA3	Activation	RARA can be recruited to GATA3 binding sites to influence GATA3 activity at its target genes[9]. (https://doi.org/10.1128/MCB.24.15.6824-6836.2004)
MYCN	RARA	Inhibition	MYCN inhibits normal RA-mediated neuronal differentiation[12] (https://doi.org/10.1186/s13073-017-0407-3)
MYCN	ZEB1		NA
MYCN	HES1	Activation	MYCN binds to the HES1 promoter and exhibits transcriptional activity[12]. (PMID: 31598396)
KLF8	GATA3		NA
KLF8	HES1		NA
KLF8	LEF1		NA
POU5F1	MYCN	Activation	MYCN and its cis-antisense gene, NCYM, form a positive feedback loop with OCT4[13]. (https://doi.org/10.1111/cas.12677)
POU5F1	SOX2	Activation	Oct4 and Sox2 bind directly to the composite sox-oct elements in both Pou5f1 and Sox2 in living mouse and human ESCs[14]. (https://doi.org/10.1128/MCB.25.14.6031-6046.2005)
ZEB1	KLF8		NA
GATA3	MYCN		NA
GATA3	SOX2	Inhibition	(indirect) MTA3 is recruited by GATA3 to repress SOX2OT transcription and then SOX2[15]. (https://doi.org/10.1016/j.isci.2019.11.009)
GATA3	KLF8		NA
KLF8	MYCN		NA
MYCN	KLF8	Activation	MYCN up-regulates PTK2; KLF8 is positively regulated by PTK2[16]. (https://doi.org/10.3390/molecules28031141)
E2F4	FOS		NA
GATA3	FOS	Activation	GATA3 binds to the promoter region of the FOS gene and activates FOS transcription[17]. (https://doi.org/10.1038/s41419-023-05888-9)
GATA3	E2F4		NA
RARA	FOS	Inhibition	RARA can directly interact with and inhibit AP-1 transcriptional activity, which includes FOS as a key component[18]. (https://doi.org/10.1210/mend.13.2.0237)
MYCN	FOS		NA
HES1	FOS		NA
GATA3	ZEB1	Inhibition	Wild-type GATA3 transcriptionally suppresses ZEB1[19]. (https://doi.org/10.2147/CMAR.S147973)
TWIST2	FOS		NA
ZEB1	POU5F1		NA

Supplementary Table 2. Model parameters of the optimized ODEs for the gene circuit governing ICM-TE transition. The rate equations for this model are provided in Equation 11 in the Methods section. Each gene i corresponds to Oct4 (x_1), Cdx2 (x_2), Esrrb (x_3) and Fgf (x_4). x(0) denotes the initial expression levels of the simulated trajectory (ICM-like state), and x(T) denotes the model-predicted expression levels at the final time point T (TE-like state). All parameters are given in arbitrary units.

**Model parameters**	x_1	x_2	x_3	x_4
$x(0)$, **Initial condition**	3.775	72.674	119.907	150.0
$x(T)$, x **at the final time point**	3.006	97.001	114.88	25
g_i, **Production rate**	1.5	17.0	75.0	20.0
k_i, **Degradation rate**	0.1	0.3	0.2	0.1
$\lambda_{1\to i}$, **Maximum Fold-change**	1.0	0.04	1.0	1.0
$\lambda_{2\to i}$, **Maximum Fold-change**	0.05	1.0	0.3	1.0
$\lambda_{3\to i}$, **Maximum Fold-change**	5.0	1.0	1.0	1.0
$\lambda_{4\to i}$, **Maximum Fold-change**	1.0	4.0	1.0	1.0
$R_{1\to i}$, **Hill threshold**	1.0	4.0	1.0	1.0
$R_{2\to i}$, **Hill threshold**	10.0	1.0	30.0	1.0
$R_{3\to i}$, **Hill threshold**	10.0	1.0	1.0	1.0
$R_{4\to i}$, **Hill threshold**	1.0	120.0	1.0	1.0
$n_{1\to i}$, **Hill Coefficient**	1.0	4.0	1.0	1.0
$n_{2\to i}$, **Hill Coefficient**	4.0	1.0	4.0	1.0
$n_{3\to i}$, **Hill Coefficient**	4.0	1.0	1.0	1.0
$n_{4\to i}$, **Hill Coefficient**	1.0	2.0	1.0	1.0
T, **Total simulation Time**	1000	1000	1000	1000

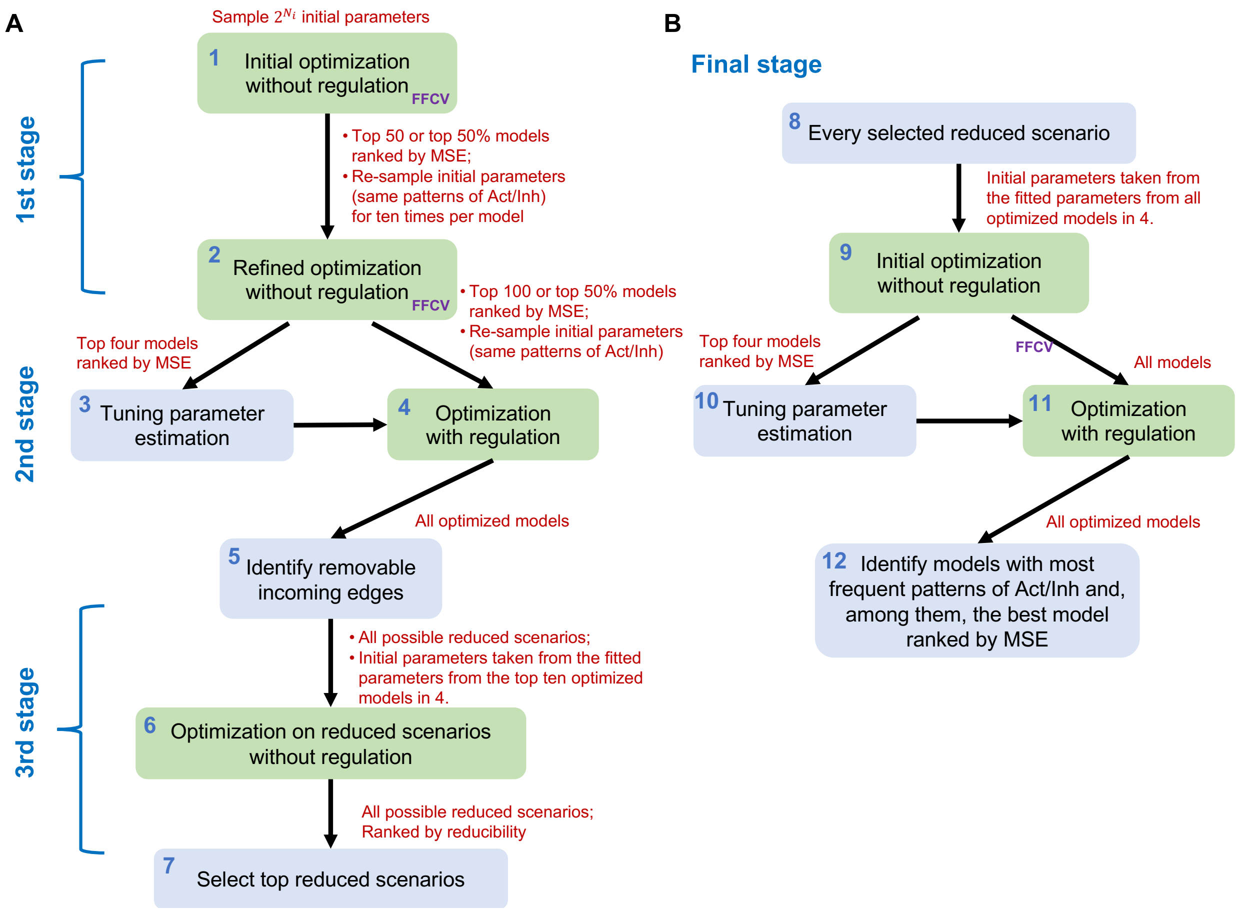

Supplementary Fig 1. Flowchart diagram for the ODE parameter optimization procedure in NetDes. The whole process consists of four stages of optimization.

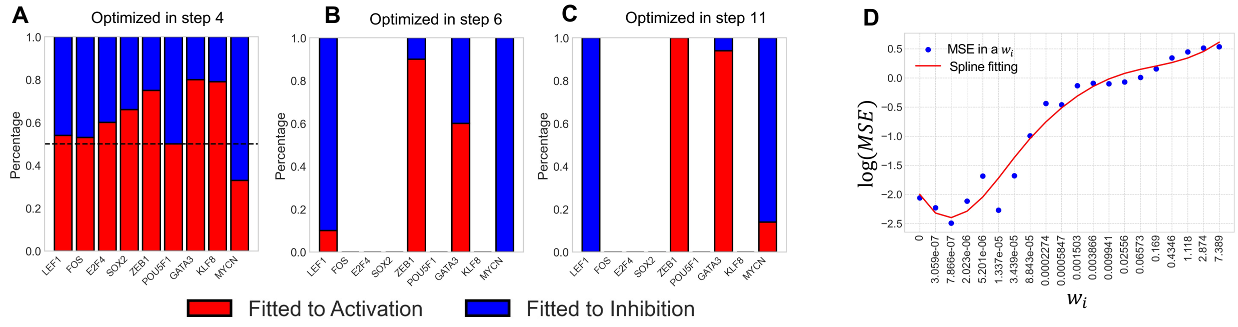

Supplementary Fig 2. Additional model optimization results for the application to iPSC differentiation scRNA-seq data. Panels **A-C** show, for each putative regulator of RARA, the percentages of optimized models inferred as activating (red) versus inhibiting (blue). These plots illustrate the outcomes at successive steps of the model optimization protocol (see **Fig. S1**): deep sampling during step 4 (A), edge removal in step 6 (B), and incorporate regulation terms in step 11(C). In later steps, the distributions are increasingly dominated by either activating or inhibiting interaction, suggesting more robust and convergent fitting outcomes. **(D)** Example of tuning parameter estimation. The scatter plot shows log(MSE) values across different values of the tuning parameter w_i (blue dots). The red curve indicates a spline fit to the data. The final value of w_i was selected as the one corresponding to the minimum log(MSE) from the curve.

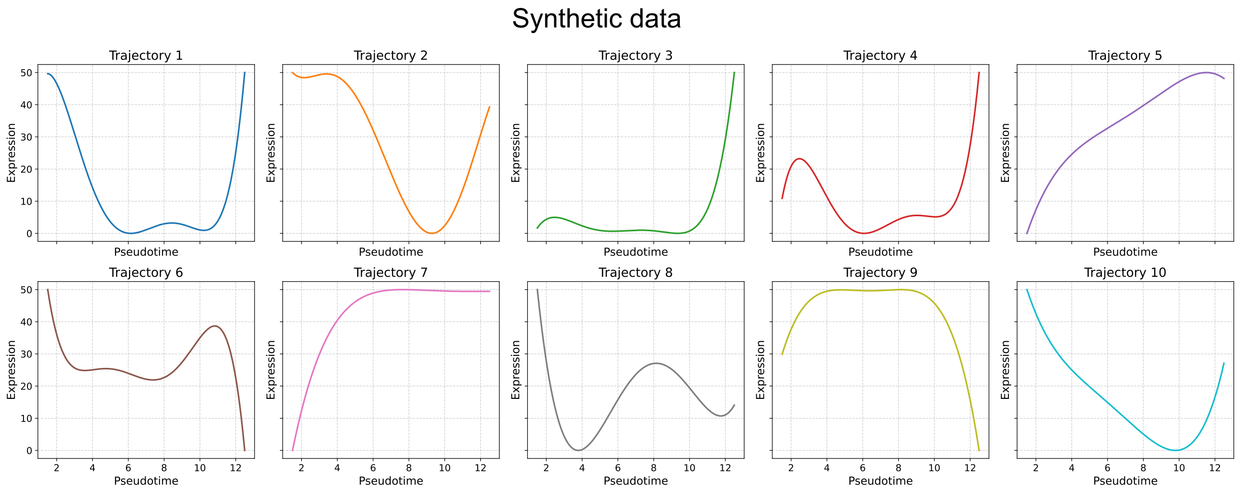

Supplementary Fig 3. Ten time trajectories for synthetic benchmarking. These trajectories were generated as described in **Supplementary Equation 1** and represent a diverse range of dynamic behaviors.

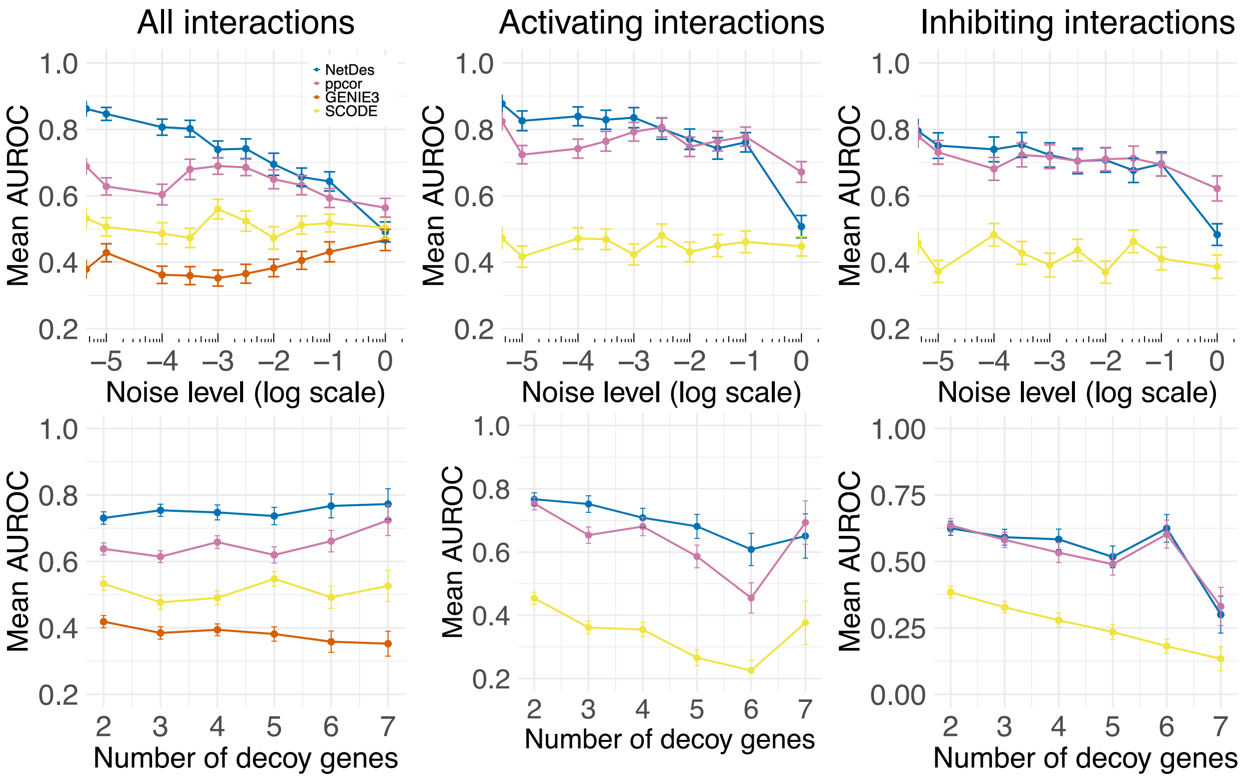

Supplementary Fig 4. AUROC results for benchmarking NetDes and other inference methods on the synthetic dataset. Top panels show the mean AUROC across different noise levels (in log scale) for all interactions (*i.e.*, both activating and inhibiting), activating interactions, and inhibiting interactions. Bottom panels show the mean AUROC across different numbers of decoy genes.

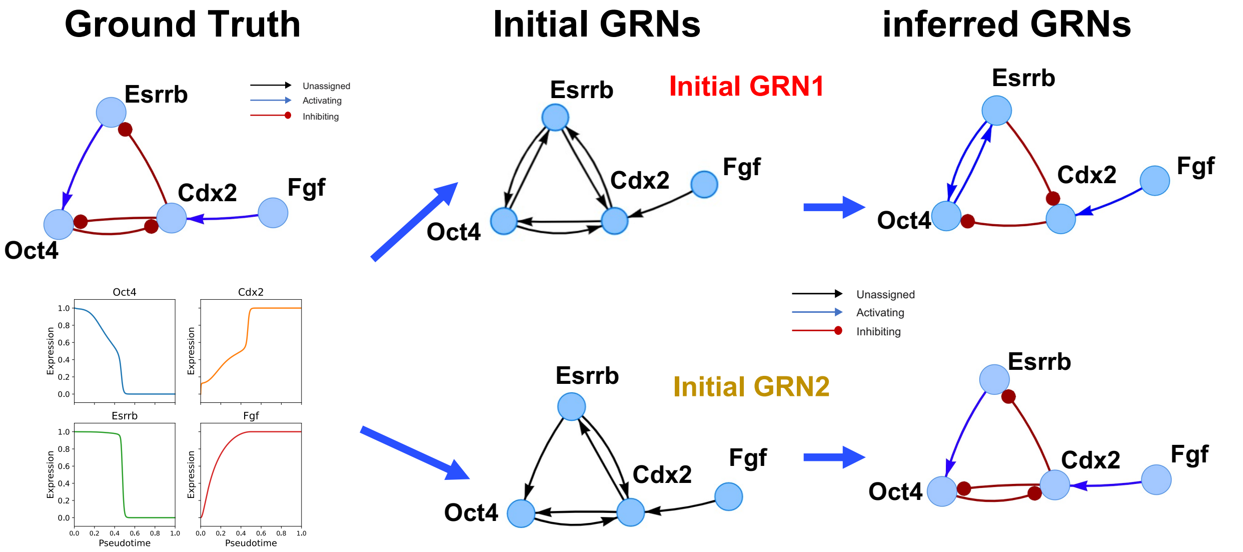

Supplementary Fig 5. NetDes inference of GRN driving cell state transition from inner cell mass (ICM) to trophectoderm (TE). The top-left panel shows the topology of the ground-truth GRN, where Fgf acts as an input node driving a three-gene circuit consisting of Oct4, Cdx2 and Esrrb. Two distinct initial GRNs (Initial GRN1 and Initial GRN2, middle panels) were provided to NetDes along with synthetic time-series expression data (bottom-left panel). The rightmost panels show the topologies of the GRNs inferred by NetDes.

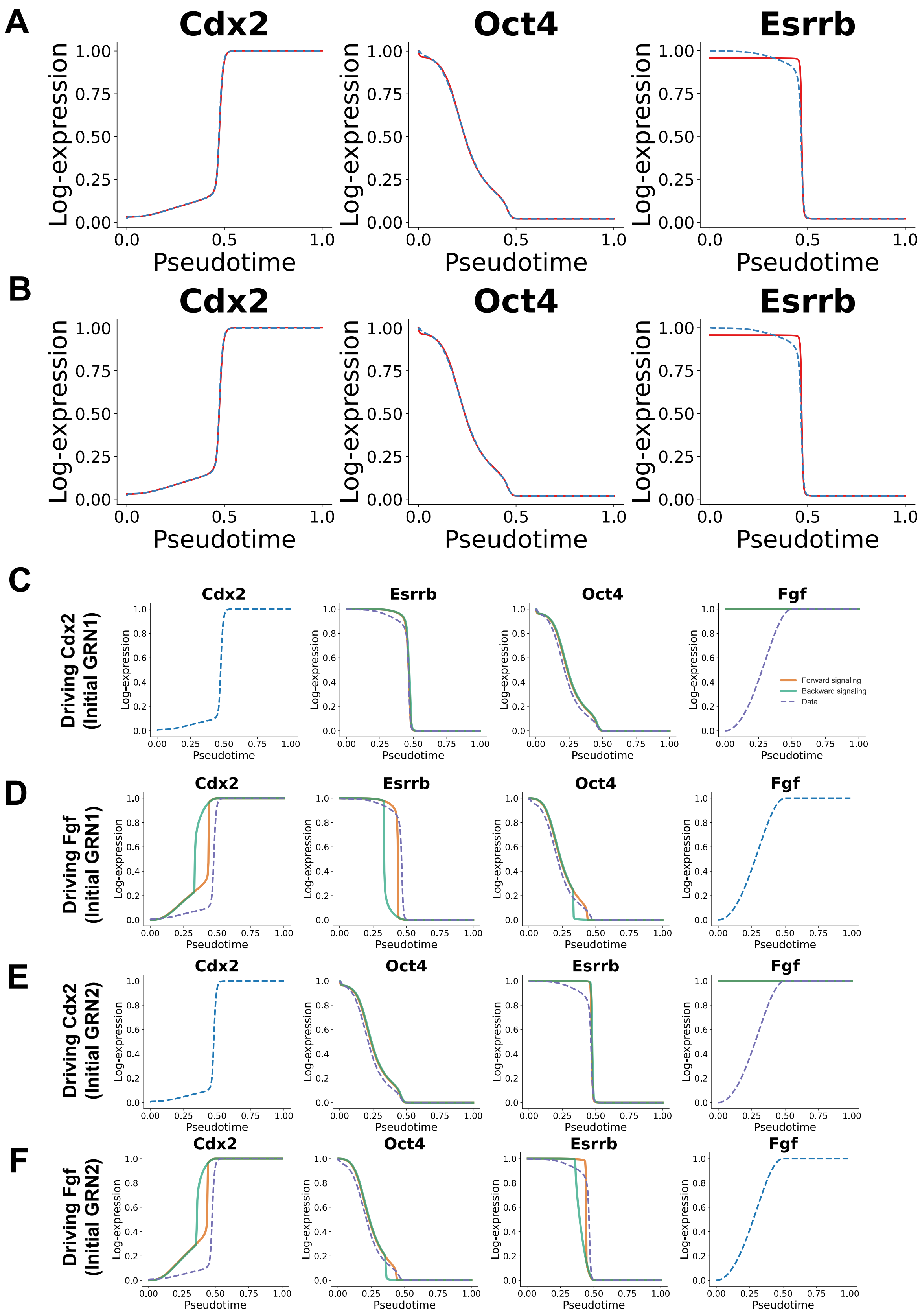
A
Cdx2
Oct4
Esrrb
Log-expression
Pseudotime
B
Cdx2
Oct4
Esrrb
Log-expression
Pseudotime
C
Driving Cdx2 (Initial GRN1)
Cdx2
Esrrb
Oct4
Fgf
Forward signaling
Backward signaling
Data
D
Driving Fgf (Initial GRN1)
Cdx2
Esrrb
Oct4
Fgf
E
Driving Cdx2 (Initial GRN2)
Cdx2
Oct4
Esrrb
Fgf
F
Driving Fgf (Initial GRN2)
Cdx2
Oct4
Esrrb
Fgf

Supplementary Fig 6. GRN simulations for NetDes inferred GRNs for the ICM-to-TE state transition. **(A-B)** Comparison of gene expression trajectories for Oct4, Esrrb and Cdx2 between the ground-truth synthetic data (blue dashed lines) and the simulated trajectories from the NetDes model (red solid lines) for the two inferred GRNs. Panel A corresponds to the upper-right GRN in **Fig. S5**, and panel B to the lower-right GRN. **(C-D)** Simulated gene expression trajectories when the first inferred GRNs was driven by Cdx2 (C) and Egf (D). Solid lines represent the simulated trajectories when the GRN was driven by the input signals along the forward (orange) and backward (green) directions. **(E-F)** Corresponding simulation results for the second inferred GRN.

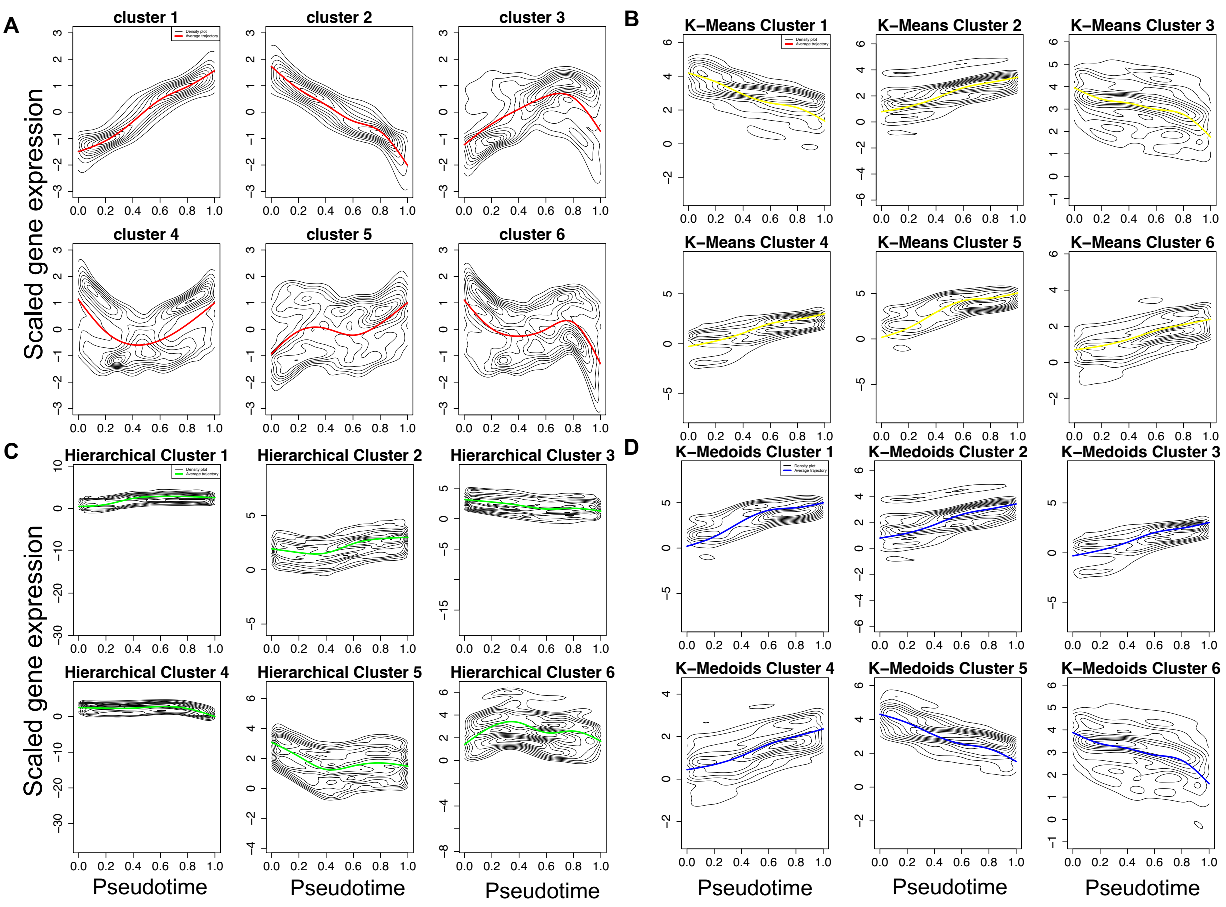

Supplementary Fig 7. Comparison of clustering algorithms on gene expression trajectories. Smoothed gene expression trajectories were grouped into six clusters by four different algorithms. In all panels, gray contour lines indicate the density of individual trajectories, and the colored curve represents the average trajectory of each cluster. **(A)** NetDes (red), **(B)** K-means (yellow), **(C)** hierarchical clustering (green), and **(D)** K-medoids (blue). Compared to the other methods, NetDes clustering yields more distinct and informative patterns in the time trajectories.

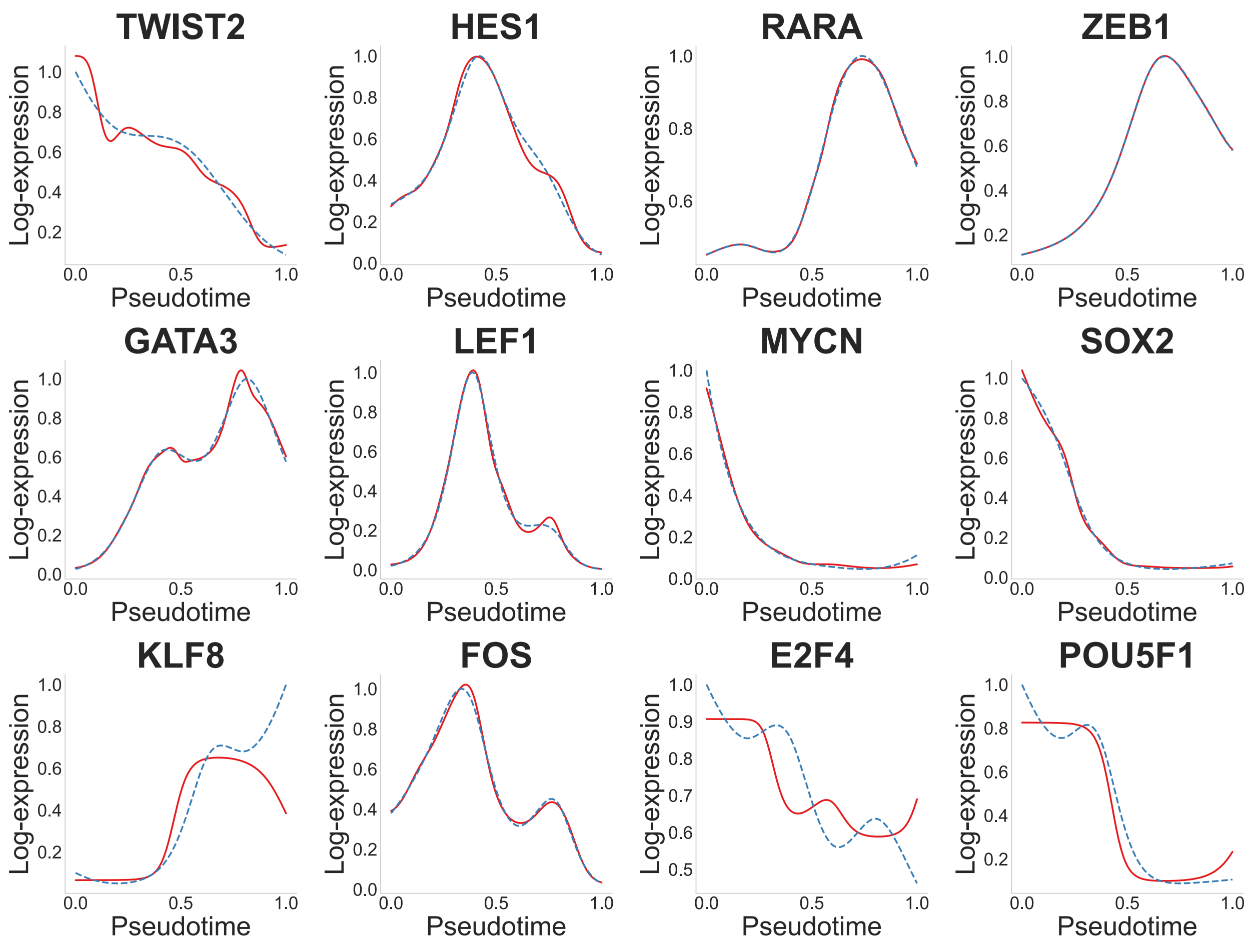

Supplementary Fig 8. Comparison between experimental and fitted gene expression trajectories for each TF. Dashed blue curves show the log-transformed, smoothed gene expression trajectories from the scRNA-seq data. Solid red curves show the simulated trajectories generated by the optimized model, using regulators' expression trajectories derived from the same scRNA-seq data.

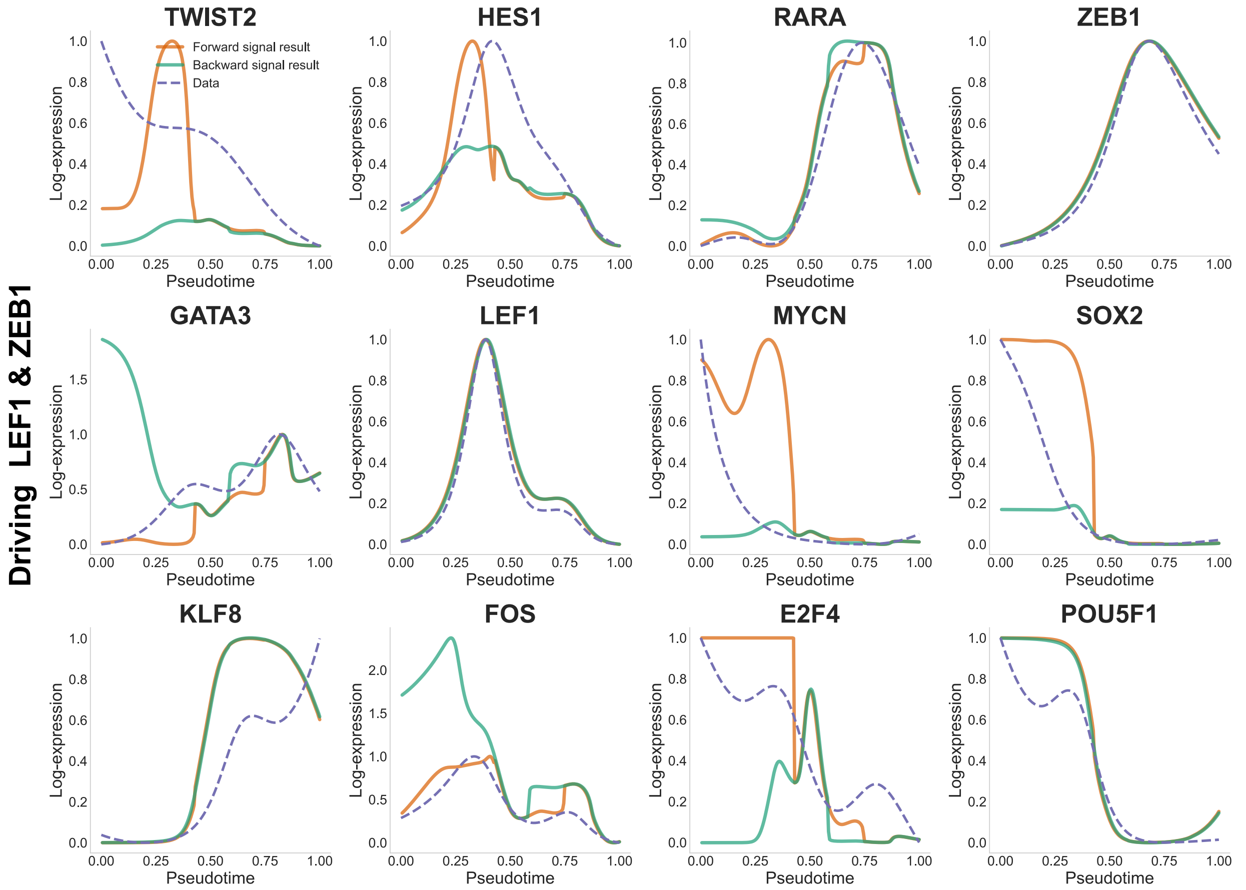

Supplementary Fig 9. Comparison of simulated gene expression trajectories when the optimized GRN was driven by both LEF1 & ZEB1. The plot is related to **Fig.4C**, with the trajectories for all genes presented here. Each plot shows the smoothed gene expression trajectories along the pseudotime (in blue dashed line), the simulated trajectories for the GRN driven by forward signaling (orange solid line) and backward signaling (green solid line).

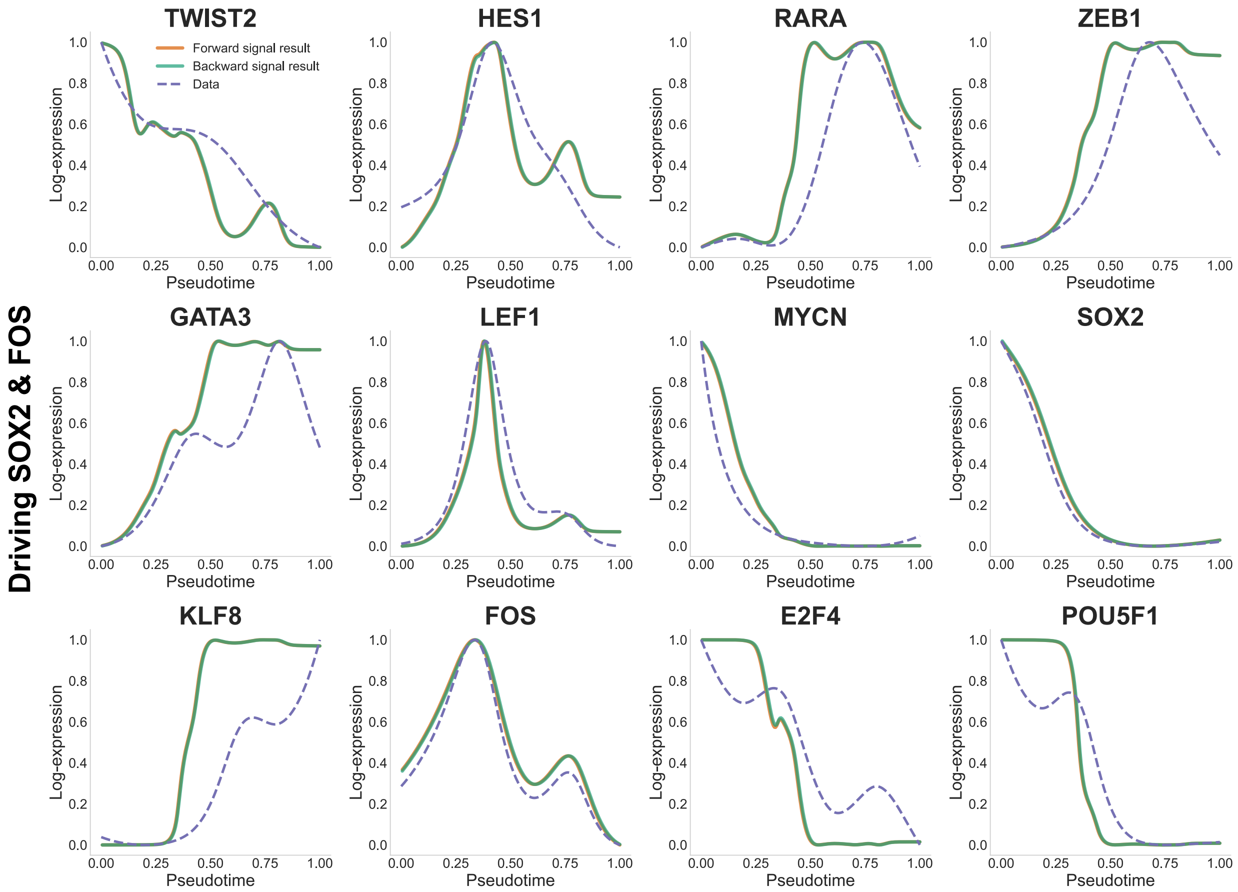

Supplementary Fig 10. Comparison of simulated gene expression trajectories when the optimized GRN was driven by both SOX2 & FOS. The plot is related to **Fig.4C**, with the trajectories for all genes presented here. Each plot shows the smoothed gene expression trajectories along the pseudotime (in blue dashed line), the simulated trajectories for the GRN driven by forward signaling (orange solid line) and backward signaling (green solid line).

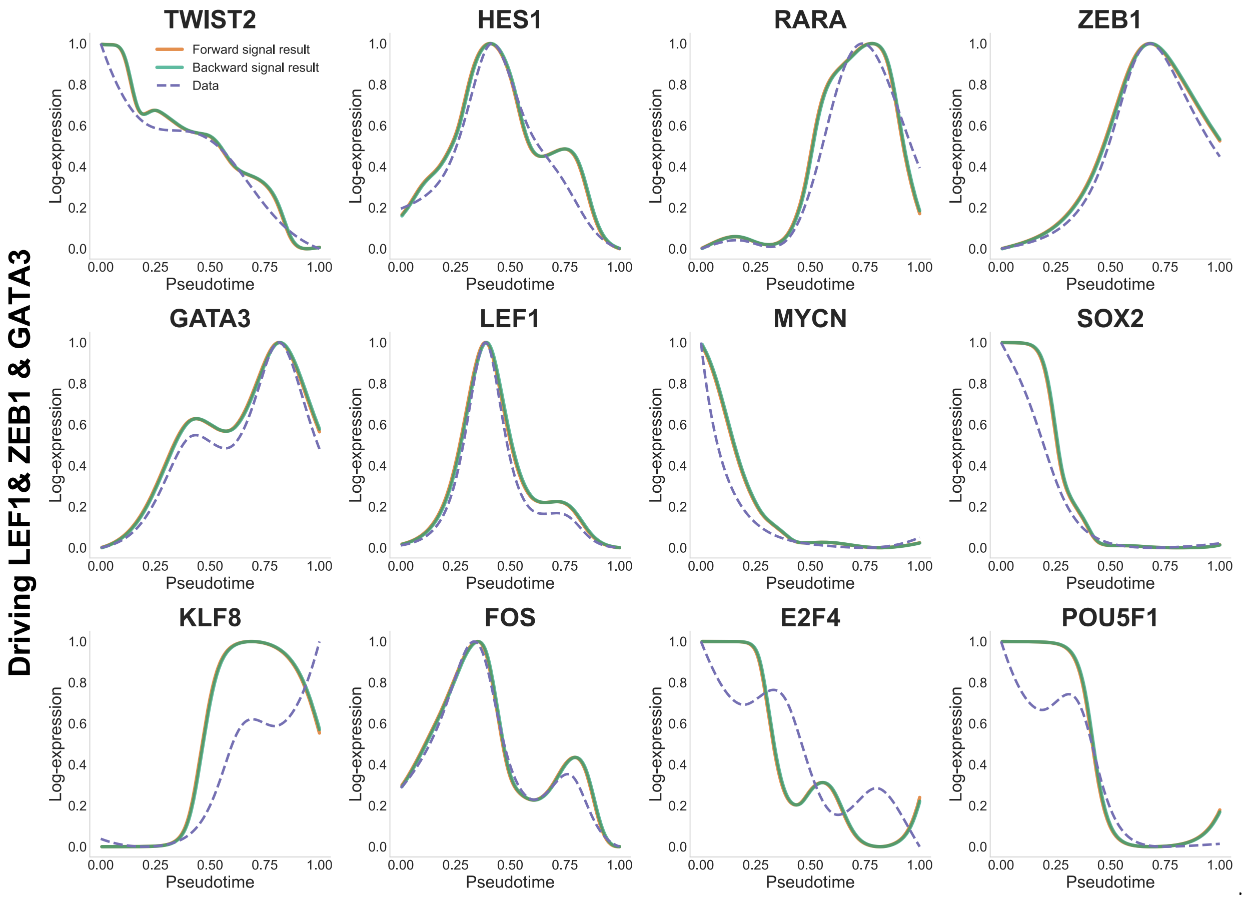

Supplementary Fig 11. Comparison of simulated gene expression trajectories when the optimized GRN was driven by LEF1, ZEB1 and GATA3. The plot is related to **Fig.4E**, with the trajectories for all genes presented here. Each plot shows the smoothed gene expression trajectories along the pseudotime (in blue dashed line), the simulated trajectories for the GRN driven by forward signaling (orange solid line) and backward signaling (green solid line).

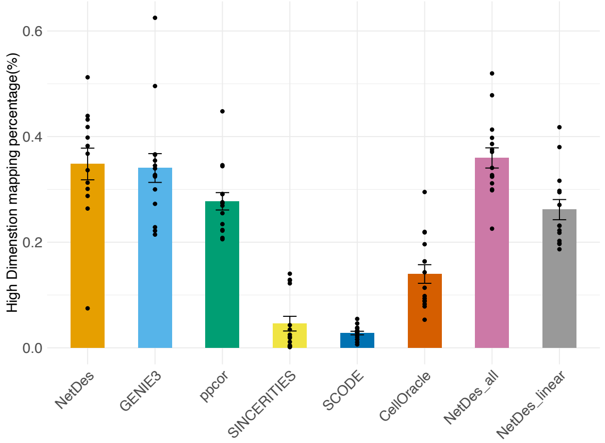

Supplementary Fig 12. Mapping percentages in full-dimensional expression space without providing initial GRN (related to Fig.5). Bar plots with scatter points showing the full dimension mapping percentages of simulated gene expression profiles to the reference profiles for each inference method.

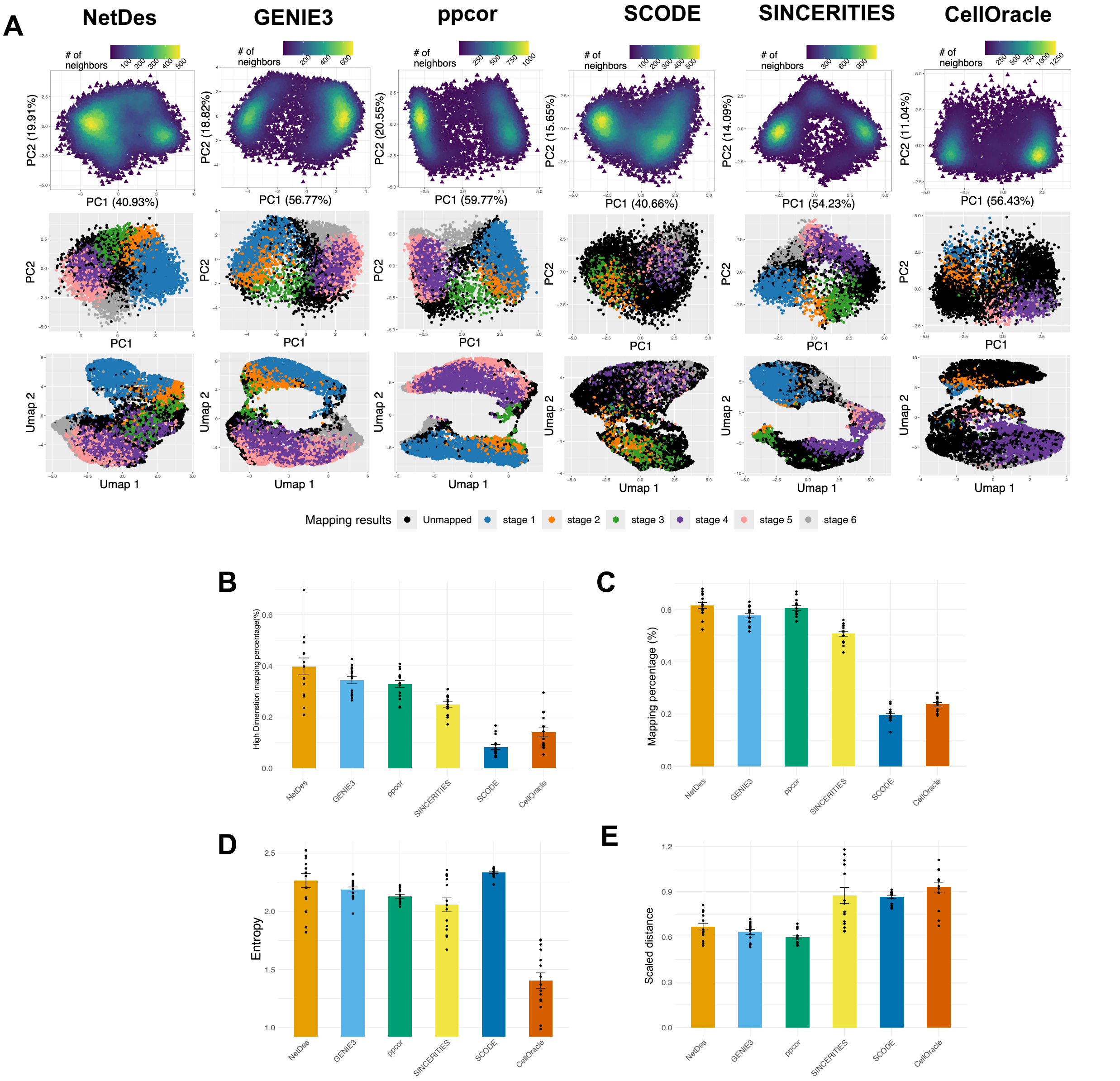

Supplementary Fig 13. Capability of GRN inference in capturing single cell gene expression states during iPSC differentiation (related to Fig.5). In this case, the same initial network as described in **Table.S1** was provided for each GRN inference method. For method other than NetDes, inferred regulatory interactions were only kept if they also present in the initial network. **(A)** Simulated gene expression profiles for the inferred GRNs with the highest mapping percentage for each method. Each set of subplots shows the density plot of simulator gene expression projected onto the first two principal components (top), the scatter plot of gene expression colored by their mapped reference states on the PCA space (middle), and the same scatter plot but projected onto the first two UMAP dimensions (bottom). **(B)** Bar plots with scatter points showing the full dimension mapping percentages of simulated gene expression profiles to the reference profiles for each inference method (CellOracle is excluded because it can't take the initial GRN as an input).

(C) Bar plots with scatter points showing the low dimension mapping percentages (PC1 – PC3) of simulated gene expression profiles to the reference profiles for each inference method **(D)** Bar plots with scatter points showing the entropy metric that quantifies the disproportion of mapped states. **(E)** Bar plots with scatter points showing the scaled distances in gene expression (see Methods for details) of unmapped simulated gene expression profiles to the nearest reference profile.

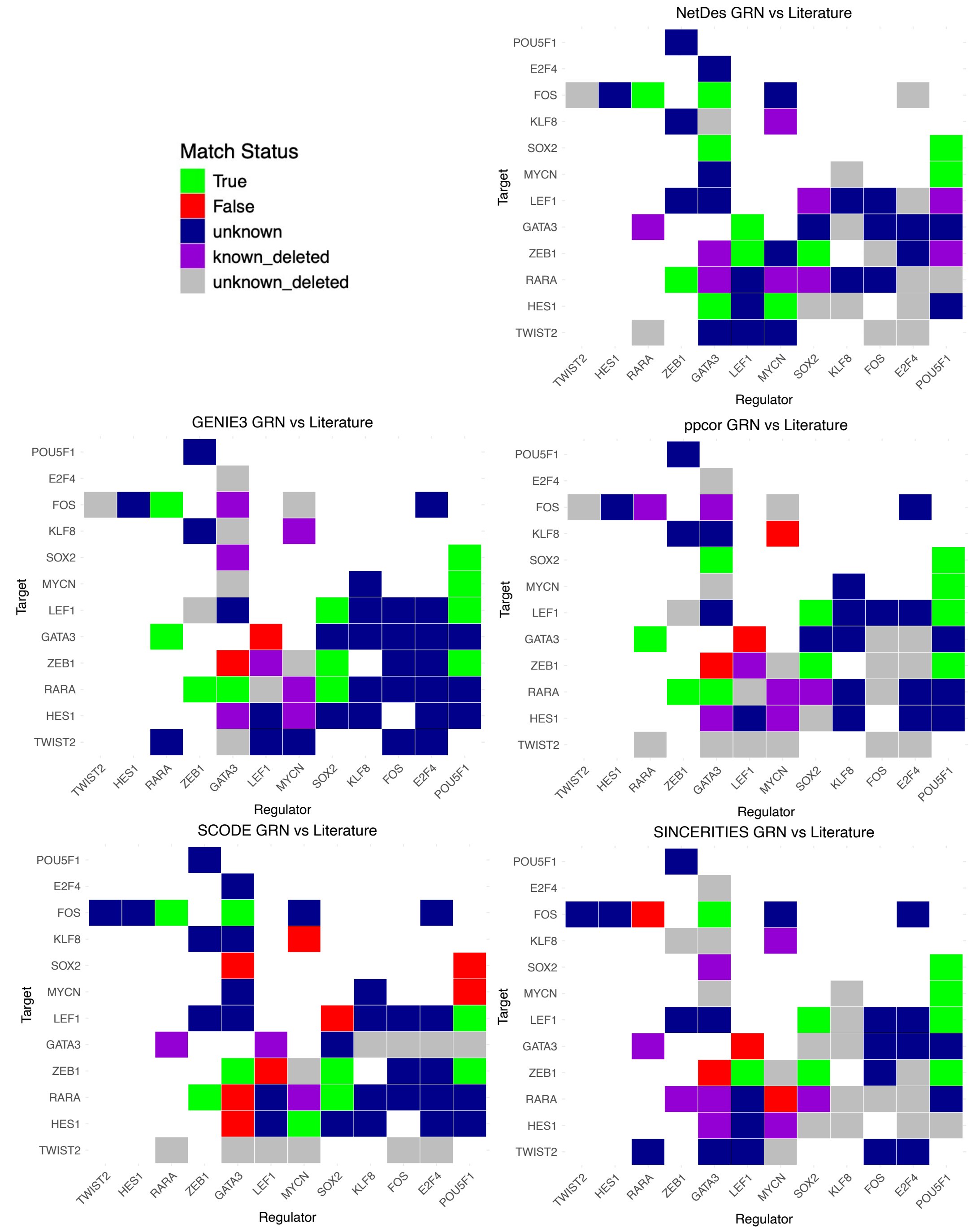

Supplementary Fig 14. Comparison of inferred regulatory edges from each method against literature evidence. This figure is related to **Fig. 5E**. For each method, we illustrated the inferred GRN with the highest mapping percentage. Each panel shows a heatmap of regulator–target interactions for each inference method.

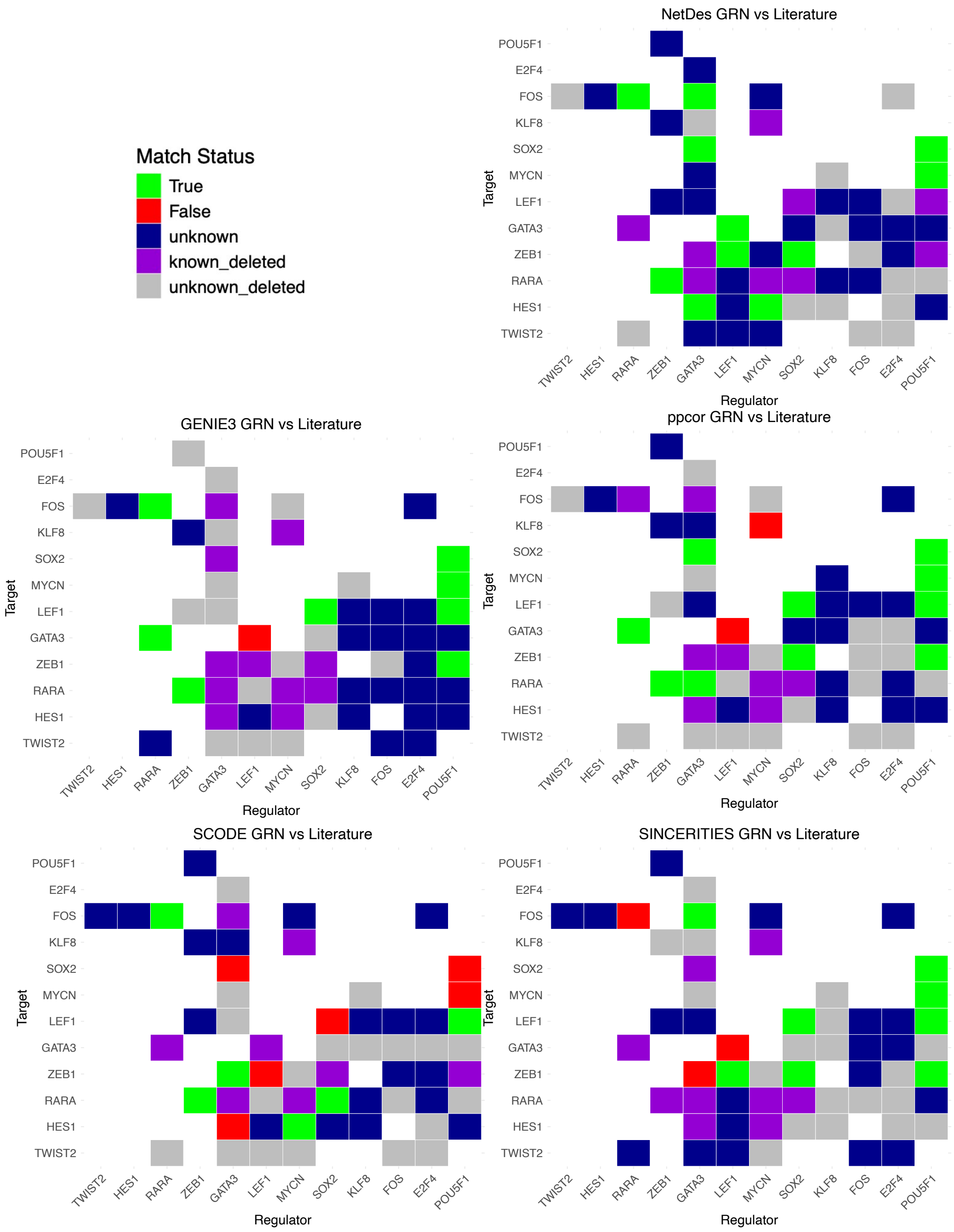

Supplementary Fig 15. Comparison of inferred regulatory edges from each method against literature evidence. This figure is related to **Fig. 5E**. For each method, we illustrated the inferred GRN with the highest accuracy according to the literature comparison.
